# In Vivo Robustness Analysis of Cell Division Cycle Genes in Saccharomyces cerevisiae

**DOI:** 10.1371/journal.pgen.0020111

**Published:** 2006-07-14

**Authors:** Hisao Moriya, Yuki Shimizu-Yoshida, Hiroaki Kitano

**Affiliations:** 1 ERATO-SORST Kitano Symbiotic Systems Project, Japan Science and Technology Agency, Shibuya-ku, Tokyo, Japan; 2 The Systems Biology Institute, Shibuya-ku, Tokyo, Japan; 3 Sony Computer Science Laboratories Incorporated, Shinagawa, Tokyo, Japan; Brandeis University, United States of America

## Abstract

Intracellular biochemical parameters, such as the expression level of gene products, are considered to be optimized so that a biological system, including the parameters, works effectively. Those parameters should have some permissible range so that the systems have robustness against perturbations, such as noise in gene expression. However, little is known about the permissible range in real cells because there has been no experimental technique to test it. In this study, we developed a genetic screening method, named “genetic tug-of-war” (gTOW) that evaluates upper limit copy numbers of genes in a model eukaryote *Saccharomyces cerevisiae,* and we applied it for 30 cell-cycle related genes (*CDC* genes). The experiment provided unique quantitative data that could be used to argue the system-level properties of the cell cycle such as robustness and fragility. The data were used to evaluate the current computational model, and refinements to the model were suggested.

## Synopsis

Robustness is a property of a system that attempts to maintain its functions against internal and external perturbations. It is one of the fundamental and ubiquitously observed system-level properties of biological systems. Understanding the cellular robustness is important, not only to gain insights in biology, but also to identify potential therapeutic targets. Robustness is estimated by measuring how much parameters can be perturbed without disrupting essential functions; comprehensive, as well as quantitative perturbations of intracellular parameters, such as gene expression, are essential for solid robustness analysis. However, the lack of experimental methodology for the comprehensive quantification and defined perturbation of parameters has prevented experimental analyses of cellular robustness. The authors developed a novel genetic screening method named “genetic tug-of-war” (gTOW) that allows systematic measurement of upper limit gene copy number. gTOW applied for the robustness analysis of cell division cycle system in the model eukaryote, *Saccharomyces cerevisiae,* and revealed the point of fragility in the system. The gTOW method is particularly suitable for systems biology research and demonstrates the value of comprehensive and quantitative perturbation experiment to uncover system-level properties of the cellular system.

## Introduction

Intracellular biochemical parameters, such as gene expression level, are considered to have become optimized through the long history of evolution so that cells can precisely perform their biological activity. These parameters must have permissible ranges against internal perturbations, such as noise in gene expression and external perturbations such as temperature variation. On the other hand, these parameters need to be dynamically changed during cellular responses against environmental changes or the cell division cycle. Recent computational analyses using mathematical models based on molecular biological knowledge revealed characteristics of these parameters, and the robustness of biological systems against parameter perturbations has been discussed [1–8]. However, little is known about the permissible ranges of parameters in real cells because there has been no experimental technique to comprehensively measure the limits of intracellular parameter.

To reduce the expression level of a target gene, gene knockout experiments, by which the expression level is reduced to zero, are used. For example, in model organisms such as *Saccharomyces cerevisiae,* comprehensive gene knockout or promoter titration analyses have been performed [9,10]. These experiments provide phenotypical information that reveals the functions of target genes. Recent synthetic knockout analyses also have provided comprehensive information on the genetic interaction of the genome [11–13]. However, such experiments do not provide quantitative information associated with the limit of expression of the target gene in order to function. On the other hand, to increase the expression level of a target gene, promoter-swapping experiments, in which the target gene's promoter is changed into a strong promoter, are used. For example, in *S. cerevisiae,* the *GAL1* promoter, which can induce strong gene expression in galactose medium, is commonly used. This method also has provided much useful information for predicting the functions of target genes, as well as genetic interactions between target genes [14–17]. However, it is also difficult to determine the upper limit of the expression of the target gene because this method ignores the native expression level and regulation of the target gene.

In this study, we attempted to estimate the upper limit of the gene expression level of each target gene by increasing the copy number of the gene. We used each target gene with its native regulatory DNA elements (promoter and terminator) as a unit so that the increased copy number of the gene can be determined quantitatively, and the gene expression level is expected to increase according to the copy number. We applied the properties of 2-micron-based plasmid with the *leu2d* marker gene, whose copy number increases more than 100 under selectable conditions. If the target gene cloned into the plasmid has an upper limit of less than 100, the plasmid copy number under the selectable condition is expected to become close to the upper limit of the target gene. We named this method “genetic tug-of-war” (gTOW).

The cell division cycle is an essential process for cells, and the process has been studied most extensively at the molecular level in S. cerevisiae. Many regulatory mechanisms of the cell division cycle in S. cerevisiae are conserved among most eukaryotic cells [18]. Recently, Chen et al. developed a comprehensive computer model of the cell division cycle in S. cerevisiae [19]. This model represents more than 100 experimentally tested phenotypes of mutants and represents and predicts some quantitative behaviors of the system [19–21]. More than 70% of the parameters in the model have a permissible range of both a 10-fold increase and decrease to maintain the cell division cycle [19].

In this study, we applied the gTOW method to evaluate the upper limit dosage of 30 cell division cycle–related genes (*CDC* genes). The upper limit data obtained were compared with other systematic quantitative and qualitative datasets to date and used to explore the relationship between the limit and the system-level property of the cell cycle. Using predictions provided by the computer model as a reference, we discussed further directions for experimental and computational studies.

## Results

### Principles of the gTOW Method

**Figure 1:**
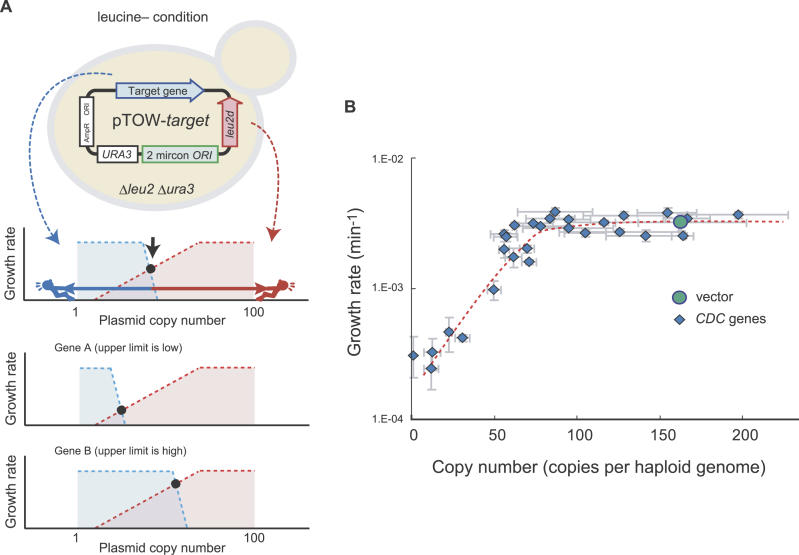
gTOW Is a Genetic Selection Method That Determines the Upper Limit Copy Number of Target Genes (A) Principle of the gTOW method. The cells with plasmid copy number close to the upper limit of each target gene are concentrated because of the genetic tug-of-war that emerges due to the high copy selection bias due to *leu2d* and the low copy selection bias due to the target gene. See text for details. (B) A scatter plot shows the correlation of maximum growth rates and plasmid copy numbers of each *CDC* gene determined in the gTOW experiment. The data used in the graphs are listed in Tables S1 and S2.

To determine the upper limit copy number of target genes, we used the genetic properties of 2-micron plasmid vectors with *leu2d*. Plasmid vectors derived from the 2-micron circle, which is a naturally observed selfish DNA in *S. cerevisiae,* have copy numbers of 10 to 40 per cell with large variations from cell to cell [22]. *leu2d* is an allele of a leucine biosynthesis gene *LEU2,* with a very weak complementation activity because it has a large deletion in its promoter. When *leu2* deletion yeast cells transformed with a 2-micron plasmid with *leu2d* are cultured under leucine− condition, the cells with higher plasmid copy numbers grow faster, and the cells with a copy number of more than 100 per cell are eventually concentrated [22]. This strong genetic selection bias was used to increase the copy number of each target gene cloned in a 2-micron plasmid with *leu2d* plasmid for genetic tug-of-war (pTOW*-target*) (Figure 1A, red arrowhead). On the other hand, if the target gene inhibits growth when it becomes more than a certain copy number (i.e., the gene has its upper limits), the cells with plasmid copy number lower than the limit grow faster. Thus, the target gene becomes another genetic selection bias toward decreasing the plasmid copy number (Figure 1A, blue arrowhead). The high copy selection bias due to *leu2d* in the leucine− condition is always constant. In contrast, the leucine− low copy selection bias due to the target gene is dependent on its upper limit, as in the case of Gene A and Gene B in Figure 1A. As a result of this tug-of-war between these two selection biases, cells with optimized plasmid copy number, which is expected to be close to the upper limit copy number of the target gene, are concentrated (Figure 1A, black arrowhead and filled circles). It should be noted that the pTOW-*target* has another marker *URA3* with complete activity, so the plasmid can be constructed and maintained in the leucine+ uracil− condition where the strong high copy bias is free. In this condition, however, there is still a weak bias toward increasing the plasmid copy number (up to about 40) due to the nature of 2-micron plasmid (see below).

### Evaluation of Upper Limit Copy Numbers of *CDC* Genes by the gTOW Method

As a test case, we analyzed the 30 cell division cycle–related *(CDC)* genes listed in Table 1 with this method. These genes are involved in the regulation of cyclin-dependent kinase (CDK) activity through the cell cycle [19,23]. We cloned each *CDC* gene with its native regulatory DNA elements, such as promoter and terminator, so that the regulation of the gene expression can be comparable to the chromosomal copy. Because these elements have not been fully determined so far, we cloned each gene with upstream and downstream DNA sequences up to the neighboring open reading frames (ORFs) into the pTOW-*target.* We measured the maximum growth rate of yeast BY4741 cells [24] transformed with each of these pTOW-*CDCgene* plasmids in the leucine− condition. We then determined the plasmid copy number (i.e., the gene copy number on the plasmid) within the cells cultured for 50 h as follows: The total DNA extracted from the cells was tested by two quantitative PCR with two primer pairs that amplify fragments of *LEU2* on the plasmid and *LEU3* on the chromosome, respectively. Then the ratio of the amount of *LEU2* to that of *LEU3* was calculated. Thus, the plasmid copy number is per haploid genome, and the actual copy number of the target gene is the plasmid copy number plus one because there is one extra copy on the chromosome.

**Table 1:**
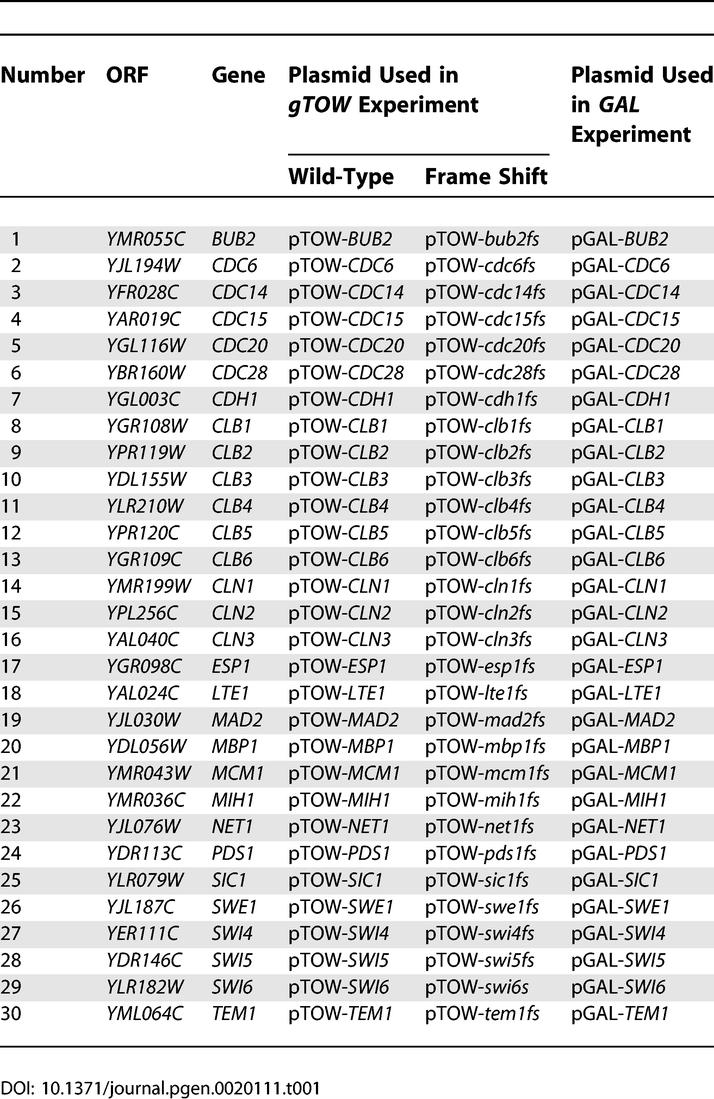
Cell Division Cycle–Related *(CDC)* Genes and Their Plasmids Used in This Study

**Figure 2:**
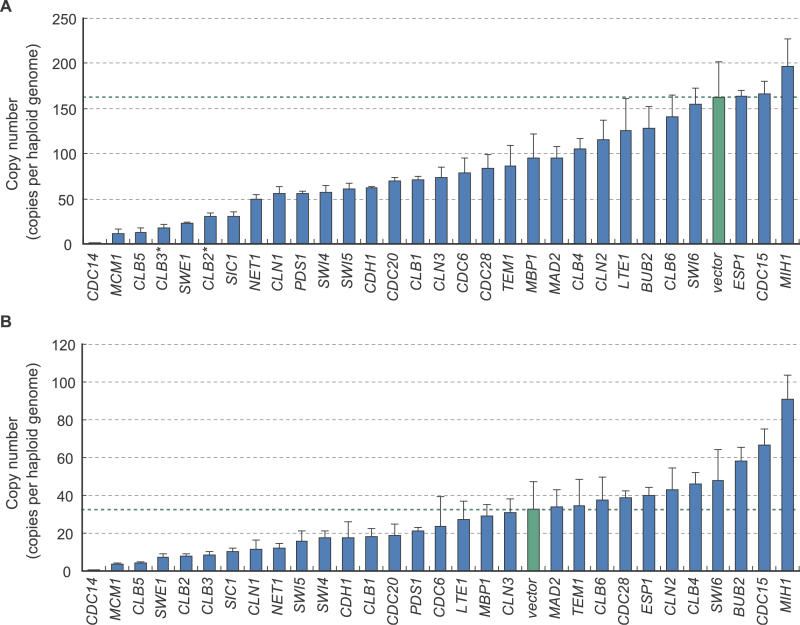
Upper Limit Copy Numbers of *CDC* Genes Copy number of the plasmid with each of the 30 *CDC* genes was determined in the gTOW experiment in leucine− (A) and uracil− (B) conditions. *The copy numbers of *CLB3* and *CLB2* were determined in the conditions with 40 μg/ml and 20 μg/ml leucine, respectively. The data used in the graphs are listed in Table S2.

Figure 1B is a scatter plot showing the relationship between the growth rate and the copy number of the 30 *CDC* genes determined in the gTOW experiment. The copy numbers and growth rates of genes with copy numbers of less than 60 showed a linear correlation. This indicates that the obtained copy numbers of these genes were determined by gTOW as shown in Figure 1A (a hypothetical mechanism for this linearity is shown in Figure S1). Genes with more than 60, but less than 80 plasmid copies, did not show obvious growth retardation, but the copy numbers are probably determined by gTOW as well (see below). Figure 2A shows the plasmid copy numbers of 30 *CDC* genes obtained in the gTOW experiment. They were diverse, from less than one to more than 100. The plasmid vector without a target gene showed about 160 copies per haploid genome. Genes with a copy number close to the vector probably do not reach the upper limit. Some cells with genes of low copy numbers showed abnormal morphologies such as cell elongation. Those morphologies were similar to the ones which have been observed when *CDC* genes are overexpressed (unpublished data), supporting the idea that the proteins encoded on the target genes were overexpressed and determined the copy number in the gTOW experiment.

**Figure 3:**
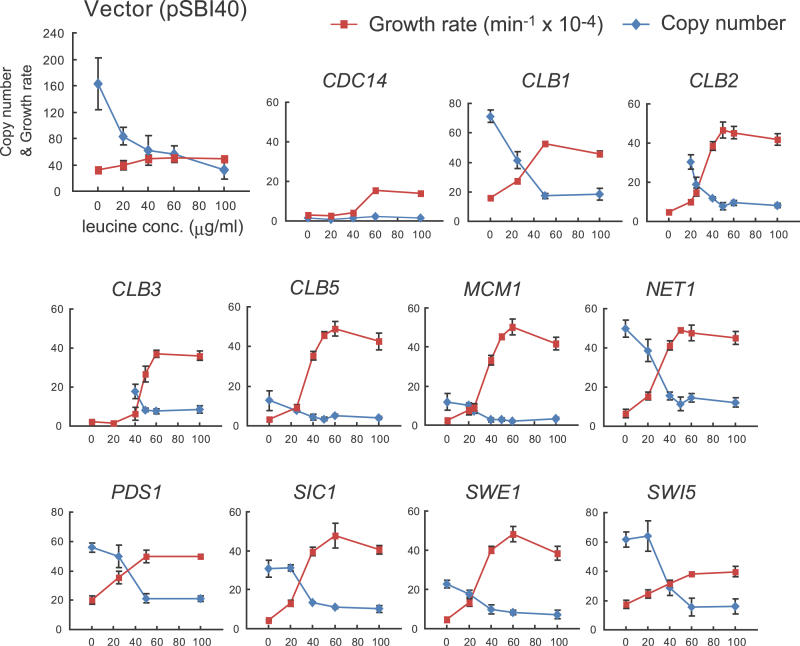
Determination of Upper Limit Gene Copy Number with Leucine Supplementation Growth and copy number of plasmids in the gTOW experiment with various leucine concentrations are shown. The data used in the graphs are listed in Table S2.

The cells with very low copy plasmid numbers (<35) hardly grew in the leucine− condition, probably because the number of *leu2d* is too low to support the *leu2* deletion. When they were cultivated for a very long time, revertant cells were sometimes observed. In this case, we further evaluated the upper limit by adding a low concentration of leucine into the growth medium in order to reduce the bias toward increasing the plasmid copy number (Figure 3). Along with the increase of leucine concentration in the medium, growth of the cells with the vector alone progressively increased, and the plasmid copy number in the cells decreased from about 160 to 40 copies per haploid genome (vector in Figure 3). We tested some genes with low plasmid copy numbers in the leucine− condition. Genes such as *CLB2, CLB3, CLB5, MCM1, SIC1,* and *SWE1* showed a dramatic switch-like decrease of growth below certain leucine concentrations (Figure 3). This switch-like decrease may be due to the nature of the regulation of these genes with positive feedbacks (see below). In these cases, we may have to regard the copy number in the leucine concentration just before the dramatic growth retardation as the upper limit.

As noted above, the plasmid copy numbers in the uracil− (leucine+) condition also seem to be determined by weak gTOW. In this condition, growth retardation due to maintaining the plasmid was not observed except *CDC14* (see below). The plasmid copy numbers, however, were rather firmly determined depending on the target genes cloned (Figure 2B), and the trend was related with the copy number trend in the leucine− condition (Figure S2). This indicates that there is a weak bias toward increasing the plasmid copy number due to the 2-micron plasmid, and the bias can be used to evaluate the upper limit of target genes under mild conditions. On the other hand, some genes such as *BUB2, CDC15,* and *MIH1* interestingly showed significantly higher copy number than the vector alone (*p* < 0.02, two-tailed Student's *t*-test). Among 30 *CDC* genes, only *CDC14* showed severe growth retardation even in the uracil− condition (*CDC14* in Figure 3). To our surprise, the copy numbers of *CDC14* through any leucine concentration were about 1 (*CDC14* in Figure 3), and the cells even in the uracil− condition were severely elongated (unpublished data). This very low upper limit counteracting the weak bias of the 2-micron plasmid probably caused the growth retardation even in the uracil− condition.

### Protein Expressed from the Target Gene Determines the Plasmid Copy Number in the gTOW Experiment

**Figure 4:**
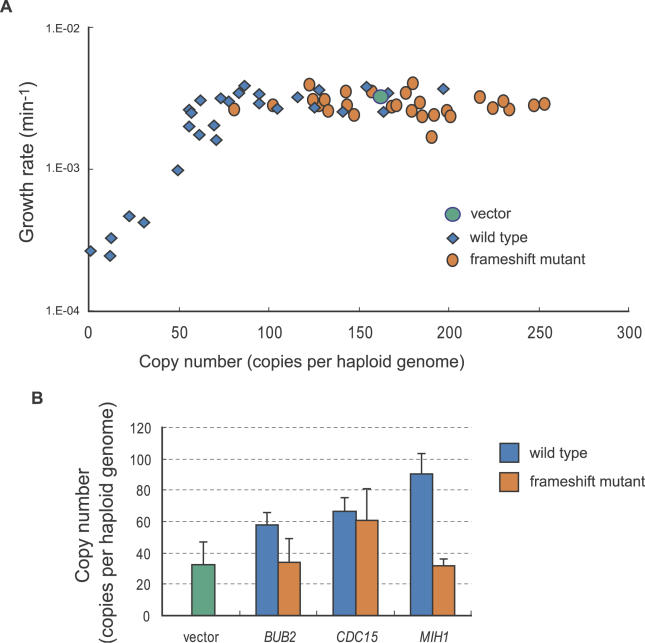
Control Experiments with Frame Shift Mutants in the gTOW Experiment (A) A scatter plot showing the correlation of maximum growth rates and plasmid copy numbers with wild-type *CDC* genes and their frame shift mutants determined in the gTOW experiment. (B) Plasmid copy numbers with wild-type and frame shift mutants in the gTOW experiment in the uracil− condition. The data used in the graphs are listed in Table S2.

We cannot exclude the possibility that the plasmid copy number in the gTOW experiment is determined because of the effect of DNA or mRNA of the target gene, because the copy number of the plasmid DNA itself is increased in this experiment. We therefore disrupted the ORFs of target genes by inserting an adenine just after the start codon to introduce frame shift mutations. Thus, the DNA sequences of the plasmids with wild-type and the frame shift mutant were exactly the same except for one nucleotide. As shown in Figure 4A, the growth rates and the plasmid copy numbers with the frame shift mutants were increased toward the levels of the vector alone. All frame shift mutants showed more than 80 copies per haploid genome, indicating that the upper limits of genes with copy numbers of less than 80 in the leucine− condition were evaluated by the gTOW experiment due to the effect of gene products (proteins) expressed from the target genes. As mentioned above, within the genes with higher plasmid copy numbers than the vector alone in the uracil− condition, *BUB2* and *MIH1* showed reduced copy numbers with frame shift mutation (Figure 4B). These genes probably work positively for rapid cell growth, most likely by ignoring some checkpoints in the cell cycle.

### The Data Obtained in the gTOW Experiment Provide Unique Information

**Figure 5:**
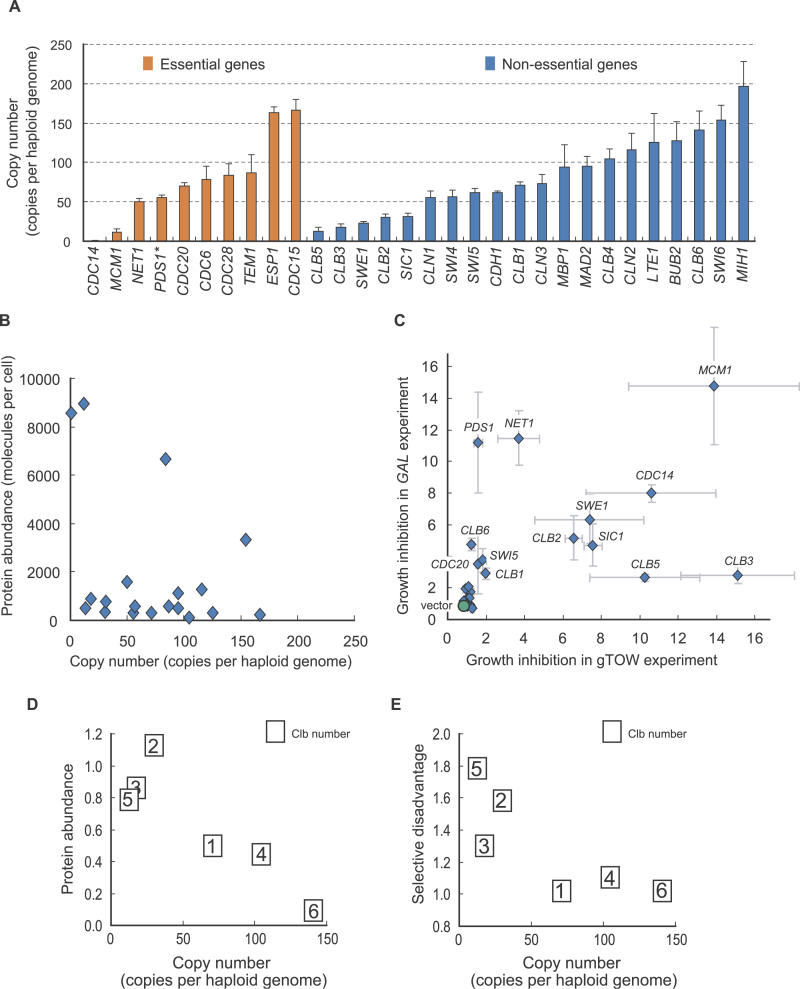
Comparisons of the Data Obtained in the gTOW Experiment with Other Qualitative and Quantitative Datasets (A) The plasmid copy numbers in the gTOW experiment with essential genes and nonessential genes [9,41]. (B) A scatter plot between protein abundance obtained by Ghaemmaghami et al. [26] and the plasmid copy number in the gTOW experiment. (C) A scatter plot of growth inhibitions between the *GAL* experiment and gTOW experiment with the 30 *CDC* genes. (D) A scatter plot between protein abundance determined by Cross et al. [21] and the plasmid copy number of B-type cyclins in the gTOW experiment. The protein abundance is the number of copies per log-phase yeast cell of the indicated cyclin protein C-terminally tagged with protein A expressed from the endogenous promoter and chromosomal location [21]. (E) A scatter plot between selectable disadvantage determined by Cross et al. [21] and the plasmid copy number of B-type cyclins in the gTOW experiment. The selective disadvantage parameter reflects differences in doubling time between wild-type and mutant cells. The data used in the graphs are listed in Tables S1, S2, and S3.

We next compared the data obtained in the gTOW experiment with other quantitative and qualitative datasets obtained in systematic analyses. First of all, the essentialities of genes [9] did not show any significant relationship with the copy number in the gTOW experiment (Spearman's *r* = 0.08, *p* > 0.05) (Figure 5A). Neither the fitness of deletion mutants of nonessential genes [9], nor haploid insufficiency [25], showed any correlation with the copy number (unpublished data). The endogenous level of proteins in wild-type cells, as determined by Ghaemmaghami et al. [26] using TAP-tagged proteins, did not show any significant correlation with the maximum tolerated plasmid copy number in our gTOW assay (Spearman's *r* = −0.36, *p* > 0.05) (Figure 5B, see also Figure S3). This indicates that the copy numbers in the gTOW experiment are determined not by the non-specific effect of overexpressed proteins to perturb general cellular functions, such as protein expression and protein degrading system, but by the specific effects of the function of each protein.

Next, we made a comparison with the standard overexpression system using the *GAL1* promoter [27]. To make the conditions the same, we constructed a series of plasmids in which each promoter on pTOW-*CDCgene* was replaced by the *GAL1* promoter (Table 1, pGAL-*CDCgene*). We then measured and calculated the maximum growth rate of the cells with the plasmid. As shown in Figure 5C, in overall, both data showed significant correlation (Spearman's *r* = 0.82, *p* < 0.01). However, some genes such as *CLB3, CLB5, CLB6, NET1,* and *PDS1* showed different degrees of inhibition between the two experiments. This is probably due to the difference of the nature of the expression system in both experiments. Thus, we found that the gTOW experiment provided unique quantitative data.

### B-Type Cyclins

B-type cyclins (Clbs) were encoded in six genes *(CLB1–6),* which are constituted of three paralogous gene pairs *(CLB1*/*CLB2, CLB3*/*CLB4,* and *CLB5*/*CLB6)* [28]. One gene *(CLB2, CLB3,* and *CLB5)* or each paralogous pair is expressed stronger and has a more major function on cellular fitness. Moreover, it is reported that the fitness of deletion mutant cells of each gene and the intracellular protein level from them have a correlation [21]. As shown in Figure 5D and 5E, *CLBs,* in particular, showed a close correlation between their copy numbers in the gTOW experiment with their intracellular protein abundance (Spearman's *r* = −0.84, *p* < 0.05) and selectable disadvantage (i.e., fitness of the deletion mutant) (Spearman's *r* = −0.77, *p* < 0.05). *CLBs* with major function (i.e., strongly expressed) had low copy numbers in the gTOW experiment, indicating that they have relatively narrow parameter ranges. Cross et al. recently measured the upper limit of *Clb2* and reported it to be less than 13-fold that of the wild-type protein amount [20]. In the leucine− condition the copy number of *CLB2* in the gTOW experiment was about 30 (*CLB2* in Figure 3). In this condition, however, the growth of cells with the plasmid was strongly inhibited. The copy number in the condition just before the strong growth inhibition (i.e., 40 mg/ml leucine) was about 12, which is almost consistent with their finding (*CLB2* in Figure 3). We also measured the Clb2 protein amount in this condition and the amount was about 12-fold that of the wild-type protein amount **(** Figure 7 **,** see below).

### Relationship between Upper Limit Gene Dosage and System Level Property of the Cell-Cycle System

**Figure 6:**
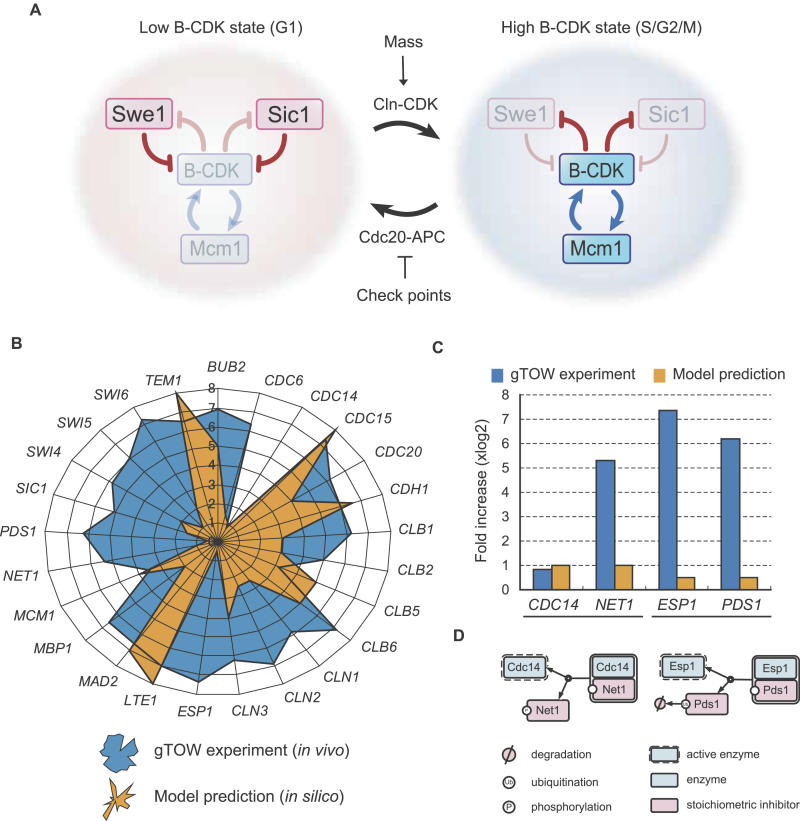
System Level Analysis of the Copy Number in the gTOW Experiment (A) Genes with low copy numbers in the gTOW experiment construct a subsystem that contains three positive feedback loops which regulate B-type cyclin activity [23,29]. This subsystem potentially makes two stable steady-states of B-type cyclin activity. Those states correspond to G1 phase and G2-S-M phase, respectively, which are altered by Cln-CDK and Cdc20-APC activity in the normal cell cycle [29]. (B) Comparison of the upper limit gene copy numbers obtained in the gTOW experiment and a computational cell cycle model. The upper limit gene copy numbers in vivo are considered as the plasmid copy numbers plus 1 in the gTOW experiment. Maximum fold variable change in xlog(2) toward the outside of the circle up to 256-fold is shown. The data used in the graphs are listed in Table S4. (C) Upper limit copy numbers of stoichiometric partners in predictions of the computer model and gTOW experiment. The data used in the graph are the same as in Figure 6B. (D) Simplified diagrams showing stoichiometric partners, where the enzymes are regulated by their inhibitors by 1:1 stoichiometric molecular interaction. The diagrams are shown as implemented in the computer model by Chen et al. [19].

To reveal the relationship between the copy numbers obtained in the gTOW experiment and properties of the cell-cycle system, we drew a map presenting current knowledge of molecular interactions in the cell cycle in S. cerevisiae and represented the copy number data on it (Figure S4). Interestingly, six out of seven genes with the lowest copy numbers were involved in the direct regulation of B-type CDK activity. They were the major B-type cyclin paralogous genes described above *(CLB2, CLB3,* and *CLB5), SIC1* that encodes a stoichiometric inhibitor of B-type CDK [19], and *SWE1* that encodes an inhibitory kinase of B-type CDK [23]. Moreover, it is known that they construct a subsystem with three positive feedback loops that regulate B-type CDK activity (Figure 6A), and these positive feedbacks make bi-stable B-type CDK states [23,29]. The dynamic alteration between the two B-type CDK states is thought to be the core process required for robust oscillation of the eukaryotic cell cycle [30]. Thus, it was first observed that the subsystem designed to dynamically change parameters conversely had a low limit of permissible parameter range. In contrast, G1 cyclins *(CLN1*, *CLN2,* and *CLN3), CDC20,* and *CDH1,* which alter the stable states, had higher copy numbers (>50).

**Figure 7:**
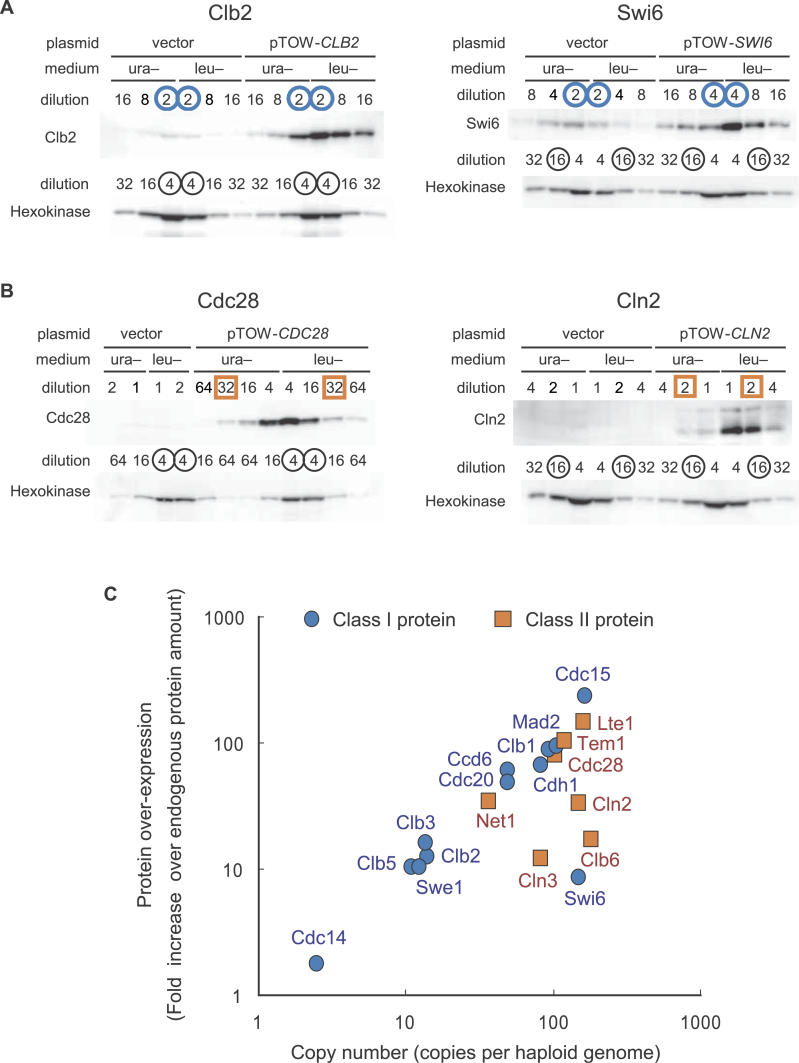
Quantification of the Protein Level Expressed in the gTOW Experiment (A) Typical example of the Western blots in Class I genes. Circled samples were used for the measurement of each protein bands. (B) Typical example of the Western blots in Class II genes. Squared samples were used for the measurement of each protein bands. (C) Scatter plot between the protein overexpression level and the gene copy number (plasmid copy number plus 1) in the gTOW experiment. The data were determined in the conditions with 100 μg/ml *(CDC14),* 20 μg/ml *(CLB2, CLB5,* and *SWE1),* and 30 μg/ml *(CLB3)* leucine conditions. The data used in the graphs are listed in Table S5.

### Comparison of the Copy Numbers in the gTOW Experiment and a Computational Cell-Cycle Model

Chen et al. constructed a computational model in which about 30 *CDC* genes in S. cerevisiae regulated CDK activity [19]. The model reconstitutes 120 phenotypes out of 131 reported phenotypes and predicts quantitative behaviors of the components [19]. We compared the prediction of upper limit copy number of *CDC* genes in this model with the ones obtained in the gTOW experiment. For the upper limits of *CDC* genes in vivo, we used the plasmid copy numbers in the gTOW experiment plus one (i.e., chromosomal copy). As shown in Figure 6B, the model generally had much lower limits than in vivo results. Moreover, the model did not reproduce the system-level characteristics, that components, which directly regulate B-type cyclins (i.e., *CLB2, CLB5,* and *MCM1*), have lower limits. In fact, in the model their upper limits were rather higher than the others, although the absolute values themselves showed rather good agreement between the model prediction and in vivo data (*CLB2, CLB5,* and *MCM1* in Figure 6B).

In the model, genes with the lowest upper limits being less than a 2-fold increase were involved in regulations by stoichiometric interactions; i.e., enzymes and their stoichiometric inhibitors (hereafter we call them “stoichiometric partners”), such as Cdc14 (protein phosphatase) versus Net1 and Esp1 (protease) versus Pds1 (Figure S7C and S7D). In the model, their regulations are easily disrupted when any of the stoichiometric partners is in relative excess more than the other, because they are regulated under the balance of 1:1 molecular interactions. Most of them did not have these extreme low limits in vivo (Figure 6C), suggesting that there are additional regulations that are not implemented into the model. However, the surprisingly low limit in *CDC14* in vivo was almost perfectly consistent with the model (Figure 6C). This probably indicates that Cdc14 activity is only regulated through the stoichiometric 1:1 interaction with Net1 as it is in the model.

### Estimation of the Overexpression of Cdc Proteins in the gTOW Experiment

In the gTOW experiment, the target gene product is supposed to be overexpressed according to the gene dosage increase. However, if transcription factors are diluted or there is a feedback regulation, it is possible that the gene copy number and the protein expression level are not linearly correlated. We thus tried to measure the Cdc proteins in the cells in the gTOW experiment with quantitative Western blot analysis. First we tried to use epitope-tagged proteins commonly used for protein detections, such as TAP-tag, myc-tag, and HA-tag [26]. However, the copy numbers of pTOW-*target,* containing *CDC* genes with epitope tags, were very different from the natural one without any tag; moreover, there was no general trend depending on the tags and protein species (unpublished data). This is probably because the tags perturbed the natural activities of the Cdc proteins. Thus, it was concluded that tagged proteins are not suitable for the perturbation experiment such as gTOW.

As an alternative method, we used specific antibodies against the target Cdc proteins provided by Santa Cruz Biotechnology, Incorporated. We used 36 antibodies against 27 Cdc proteins tested in the gTOW experiment in this report (Table 2). Among them, 12 antibodies could detect endogenous target Cdc proteins in Western blot analysis. The typical examples were Clb2 and Swi6 shown in Figure 7A (the others were shown in Figure S6A). We classified these antibodies and detected proteins as Class I. The overexpression of each Class I target protein in the gTOW experiment was measured as a fold increase over the endogenous target protein (Table S5). The other seven antibodies could only detect the overexpressed target proteins in the gTOW experiment. The typical examples were Cdc28 and Cln2 shown in Figure 7B (the others were shown in Figure S6B). We classified these antibodies and detected proteins as Class II. The overexpression of each Class II target protein in the gTOW experiment was estimated as a least-fold increase over the endogenous target protein according to the serial dilutions of the protein sample (Table S5). Thus, the estimated overexpression of each Class II protein should be less than the real one. The others (17 antibodies) could not detect the target protein in our system. We classified these antibodies as Class III.

**Table 2:**
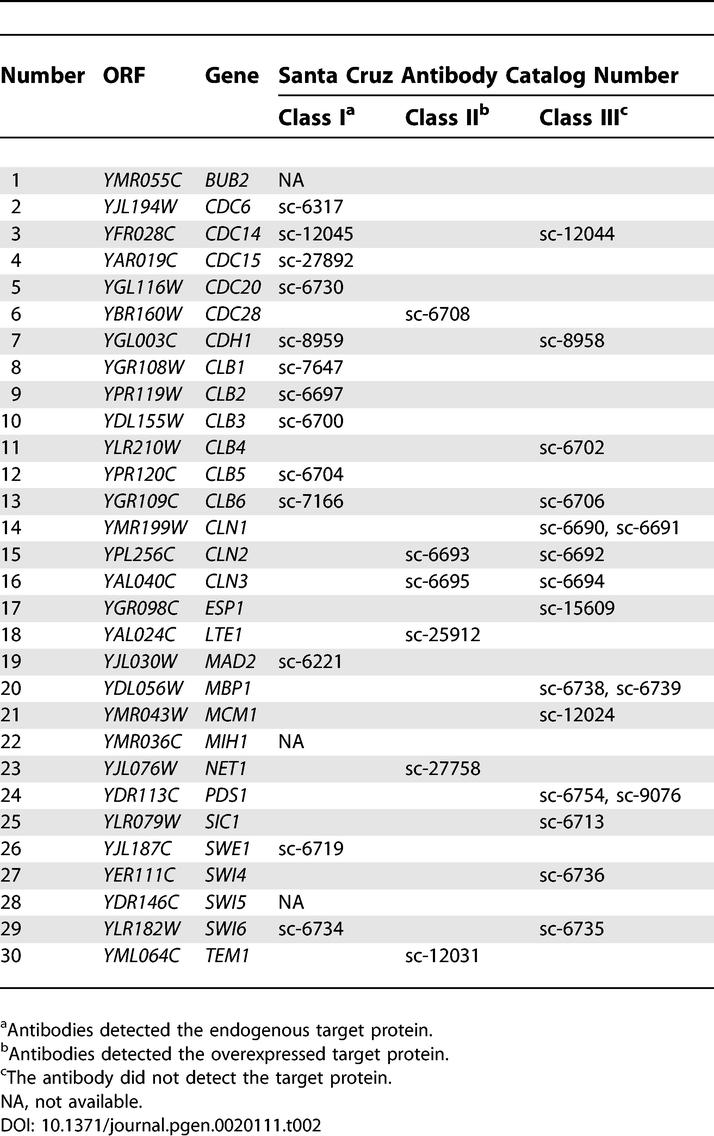
Antibodies Used for the Detection of Cdc Proteins.

Figure 7C is a scatter plot showing the relationship between the protein overexpression and the gene copy number of the *CDC* genes in the gTOW experiment. The overexpression of Class I protein, except Swi6, and the least overexpression of some Class II proteins (Cdc28, Lte1, Tem1, and Net1) showed a significant correlation with their gene copy numbers (Pearson's correlation coefficient, *r* = 0.94). Although Swi6 is overexpressed in the gTOW experiment (8.48-fold), the level was apparently inconsistent with the gene copy number (149 copies). We thus confirmed that in all cases we tested, the target Cdc proteins were overexpressed in the gTOW experiment, and in most cases the levels were correlated with the their gene copy number.

## Discussion

In this study, we reported a genetic screening method that evaluates the upper limit copy number of target genes. In this method, we used a gene with its native promoter as a unit to evaluate the upper limit copy number to inhibit cellular growth, so that we could quantitatively and directly compare the upper limits among various genes.

Principally, the gTOW experiment causes overexpression of target genes. In *S. cerevisiae,* the *GAL1* promoter system is the common way to overexpress target genes [27]. We compared the growth inhibition in the gTOW experiment and *GAL1* promoter expression system, but the data did not show complete positive correlations (Figure 5C). The differences between the two experimental results are explained by the native expression level and the expression regulation of each target gene. If the native expression level of the target gene is high, the expression from *GAL1* promoter does not cause so much “overexpression,” but increasing the copy number in the gTOW experiment does cause overexpression. For example, *CLB3* and *CLB5* did not show strong growth inhibition in the *GAL* experiment but did in the gTOW experiment (Figure 5C); probably because they have higher native expression levels [21] (Figure 5D). *CLB6* and *PDS1* are the opposite case [21,26]. In addition, if there are transcriptional and translational regulations, such as periodic expression during cell cycle and feedback regulation within the target gene, the results of both experiments will be different. Recently, Sopko et al. reported a comprehensive overexpression analysis using the *GAL1* promoter [17]. Their results and the results obtained in our *GAL1* experiment showed little accordance in growth inhibition (unpublished data). One of the reasons for this may be the existence of GST-tag in their experiment. In fact, we observed that the copy numbers in the gTOW experiments were perturbed by the commonly used epitope tags (unpublished data). Thus, when determining the quantitative effect of a target protein, a tagged protein is not preferred. Thus, the gTOW experiment enabled systematic evaluation of the upper limit of gene expression, about which little has been known hitherto.

The copy numbers of the 30 *CDC* genes determined in the gTOW experiment were very diverse, ranging from 1 to more than 100 (Figure 2A). The data revealed some interesting properties of the cell division cycle in *S. cerevisiae,* which have been very difficult to clarify. Six out of the top seven genes with the lowest copy numbers constitute a subsystem that regulates B-type CDK activity, which was a core process in the cell cycle with very dynamic properties (Figure 6A). We speculate that because the parameter range should be tuned up to be narrow in a system with very dynamic properties, the system should show high fragility against perturbations that change the quantity of parameters as a trade-off of the dynamics. In other words, the gTOW experiment was very effective to reveal dynamic subsystems that are fragile to changes in parameter.

To our surprise, *CDC14* has a very low upper limit of less than two copies per haploid genome (i.e., just one extra copy other than the chromosomal one). Interestingly, this extreme low limit was almost consistent with that predicted by the computer model developed by Chen et al. (Figure 6C), which could be explained by the 1:1 stoichiometric inhibition by Net1 [31,32]. However, other stoichiometric partners, Esp1 and Pds1, did not show such extreme low limits, although the model also predicted very low limits (Figure 6C). The discrepancy is probably explained by the fact that Esp1 needs to be recruited into the nucleus by Pds1 for its full function [33], the regulation of which is not yet implemented into the model. If there is such regulatory mechanism in a system, the system should be rather robust even when the stoichiometric balance is perturbed. We do not yet understand why the *CDC14* regulation evolved to be extremely fragile against the amount of change, but it might be a trade-off of some properties of the subsystem that *CDC14* is involved.

We used the data obtained in the gTOW experiment to evaluate a computer model. Generally, because intracellular biochemical parameters are very difficult to determine, it is difficult to evaluate the parameters in computer models. Since permissible ranges of parameters in a model are the integrative result of the network structure and parameters in the model, the parameter ranges are a very useful measure to evaluate the model's correctness and to suggest the direction to improve it [6]. The models by Chen et al. showed much fragility relative to the gTOW experimental data. We suggest two major issues to be improved in the model: one is stoichiometric partners as mentioned above and the other is paralogous gene pairs. The model implemented only one of each paralogous gene pairs (i.e., *CLB2* of *CLB1*/*CLB2, CLB5* of *CLB5*/*CLB6,* and *CLN2* of *CLN1*/*CLN2*), but each of the paralogous gene pairs had very different copy numbers (i.e., upper limits) between them in the gTOW experiment (Figure S5). The issue of how paralogous gene pairs are involved in cellular robustness is still being argued [34–36] and it will be very interesting to test how robust the model becomes when the paralogous genes exist.

The gTOW method may also be used for genetic screening of positive growth regulators. Under mild copy number–increasing bias (i.e., uracil− condition), *MIH1* and *BUB2* had significantly higher copy numbers than the vector (Figure 4B). Mih1 is known to dephosphorylate inhibitory phosphorylation of B-type CDK in Tyr-19 residue [23]. In *S. cerevisiae,* phosphorylation in Tyr-19 is involved in the morphological checkpoint [23]. High copy number *MIH1* may cause faster growth ignoring the morphological checkpoint. Interestingly, overexpression of Cdc25, the human homolog of Mih1, is known to be closely related to cancer development [37], part of which might be related to factors in cancer development as in the case of *MIH1*. Positive growth factors, which can be identified by gTOW experiments, under mild bias, potentially contain factors related to cancer development such as *MIH1*.

We estimated the protein overexpression of about 19 Cdc proteins out of 30 tested in the gTOW experiment and confirmed that most of the Cdc proteins tested were overexpressed with a good agreement with their gene copy numbers (Figure 7C). The overexpression of 12 Cdc proteins was appropriately measured with endogenous protein level (referred to as Class I proteins in this study, Figure S6A and Table S5). These will be good resources for further precise quantitative analysis, such as the protein expression in synchronized cells, or single cell variation in the gTOW experiment. Among Class I proteins, only Swi6 showed apparent discrepancy between protein overexpression level and gene copy number in the gTOW experiment (Figure 7C); the mechanism how this occurs is also an interesting future issue. Current epitope tags used for protein detection were not preferable for the perturbation analysis such as gTOW (unpublished data), but specific antibodies were not comprehensive or qualitative enough; we thus need more comprehensive and qualitative technology to detect proteins with lowest perturbations.

In conclusion, using the gTOW method, we obtained upper limit gene copy numbers, about which little has been known at a system-wide level. This sort of quantitative data represent how intracellular parameters are set up in a certain biological system, and thus represent the robustness and fragility of the system against internal perturbations, which have been very difficult to assess with experimental data. In addition, as shown here, the data can be used to evaluate computer models and to improve them. The gTOW method can also be applied to genome-wide analysis of upper limit gene copy numbers, other than those of *CDC* genes, as well as to profiling of quantitative genetic interactions in mutant strains.

## Materials and Methods

### Yeast strains and growth conditions.

A yeast strain BY4741 *(MAT*a, *his3Δ1, leu2Δ0, met15Δ0,* and *ura3Δ0)* [24] was used in this study. Yeast cells were cultured in SC media [38] supplemented with indicated amino acids (uracil, leucine). 2% glucose was used as a carbon source, except in the *GAL* experiments.

### Plasmid constructions.

The plasmids constructed and used in this study are listed in Table 1. PCR was done with KOD-Plus high fidelity DNA polymerase (TOYOBO, Japan) according to the manufacturer's protocol. pSBI40 is a pYEX4T-1 derivative [39] (the full nucleotide sequence is available upon request). pSBI104 used in the *GAL1* promoter-driven overexpression experiments was made by replacing *CUP1* promoter and GST in pSBI40 by gap-repair [40] with PCR fragment containing *GAL1* promoter amplified from genomic DNA using primer OSBI0246 and OSBI0247 (primer sequences are listed in Table S6B). For the *gTOW,* according to the *Saccharomyces* Genome Database (SGD) [41], DNA fragments containing target *CDC* genes (listed in Table 1), with upstream and downstream sequences up to their neighboring genes, were amplified from genomic DNA of BY4741 by PCR using primer sets (“up primer” and “down primer” for each gene, listed in Table S6A), then cloned into pSBI40 by the gap-repair method in BY4741. The constructed plasmids were named pTOW-*target*. For overexpression experiments from the *GAL1* promoter, DNA fragments containing target cell-cycle genes from ATG to their downstream end were amplified from the genomic DNA by PCR using primer sets (“*GAL* primer” and “down primer” for each gene, listed in Table S6A), then cloned into pSBI104 by the gap-repair method in BY4741. The constructed plasmids were named pGAL-*target*. To make frame shift mutation, adenine was inserted just after the ATG codon of each gene except *CDC14* (adenine was inserted into + 23 nt from the ATG codon), *CDH1* (cytosine was inserted +128 nt from the ATG codon) and *PDS1* (adenine was inserted +7 nt from the ATG codon); two PCR fragments amplified from each tug-of-war plasmid construct were amplified using: (1) “frame shift forward primer” for each gene and OSBI159 and (2) “frame shift reverse primer” for each gene and OSBI160; then they were combined and cloned into pSBI40 by gap-repair (primer sequences are listed in Tables S6A and S6C). The constructed plasmids were named pTOW-*targetfs*. Plasmids with more than two independent isolates from each plasmid construction were recovered from yeast, and the structure was checked by restriction digestion and partial nucleotide sequencing.

### gTOW procedure.

BY4741 cells with pTOW-*target* containing each target gene (listed in Table 1) were grown in SC without uracil, and then they were transferred into SC without leucine and uracil. The optical density of 595 nm of the culture was monitored using a microplate reader (Model X680 Bio-Rad, Hercules, California, United States) at 30 °C without agitation every 30 min for 50 h. Mean doubling time (min) at maximum growth rate (min^−1^) during the 150-min interval from at least three independent experiments was calculated. For the *GAL* experiment, BY4741 cells with pGAL-*target* containing each target gene (listed in Table 1) were grown in SC without uracil, before being transferred to SC without uracil with 2% galactose.

The plasmid dosage in a yeast cell was determined by two kinetic PCRs using total DNA extracted from yeast cells as a template. Yeast cells collected from 200 μl of saturated culture were suspended in lysis solution (10 mM Na-phosphate [pH 7.5], 1.2 M sorbitol, and 2.5 mg/ml Zymolyase 100T) (Seikagaku, Japan) and incubated for 10 min at 37 °C to digest the cell wall. Then the cell suspension was treated at 94 °C for 15 min, at −80° C for 5 min, then at 94 °C for 15 min. The cell suspension was chilled and centrifuged. Supernatant (containing total DNA) was used for the following two kinetic PCRs: For the kinetic PCR, we used LightCycler FastStart DNA Master^PLUS^ SYBER Green I (Roche, Germany) with LightCycler 2.0 instrument (Roche), according to the manufacturer's protocol. Supernatant (2 μl) was mixed with each reaction mix (18 μl) containing 0.5 μM of *LEU2* (*LEU2*-F and *LEU2*-R) and *LEU3* primer sets (*LEU3*-F and *LEU3*-R); primer sequences are listed in Table S6D. *LEU2* and *LEU3* primer sets were used to amplify and quantify *LEU2* genes from the plasmid and *LEU3* gene from the genome, respectively. By comparing the relative quantity of *LEU2* gene and *LEU3* gene, the copy number of the plasmid per haploid genome (BY4741 is a haploid strain) was estimated. The calculation is: plasmid copy number = 2^(I.P._*LEU3* - I.P_*LEU2*)^ *,* where I.P._*LEU3* and I.P._*LEU2* are the PCR cycle numbers at inflection points of the PCR amplification curves of the *LEU3* and *LEU2* genes, respectively. Strictly speaking, the number determined by this procedure is not equivalent to the copy number per cell, because in G_2_-M phase cells, there are two copies of genome per cell. Thus, the copy number is per haploid genome. We calculated the gene ratio of wild-type S288C strain (with *LEU2* and *LEU3* on the chromosome) with eight independent experiments (1.07 ± 0.16). The host strain BY4741 gave 5 × 10^−4^, confirming that BY4741 is a yeast strain with *leu2* deletion *(leu2Δ0)* [24]. For each experiment in this study, we determined the copy number of more than three independent isolates from each plasmid construction, and calculated the mean value with standard deviation.

### Quantification of Cdc protein overexpression.

Cells with vector pSBI40 and pTOW-*target* were cultivated in 2 ml SC without uracil and 2 ml SC without leucine (otherwise stated) for two overnights, then 4 ml of the fresh medium were added to the culture which was then cultivated for 4 more h to refresh the cells. DNA was extracted from the cells and the plasmid copy number was determined. Protein extraction was performed as described [42]. Briefly, cells from each 2-ml culture were collected and suspended in 400 μl of 0.2 N NaOH; after 5 min of incubation at room temperature, cells were collected and re-suspended in 100 μl of SDS-sample buffer [42], then heated at 100 °C for 5 min. Cell debris was removed by centrifugation, then the supernatant with indicated dilution was separated in 10% SDS-PAGE for the standard Western blot procedure.

In immunoblotting, each target Cdc protein was detected using its specific antibody (listed in Table 2, obtained from Santa Cruz, California, United States) with 1/200 dilution, and anti-Goat IgA peroxydase conjugate (A5420, Sigma, St. Louis, Missouri, United States) with 1/5000 dilution. Hexokinase was detected using anti-hexokinase antibody (100-4159, Sigma) with 1/1000 dilution, and anti-rabbit IgG, IgM, IgA HRP conjugate (SAB1003, Open Biosystems, United States) with 1/5000 dilution. Detection of immune complex was done with SuperSignal West Dura Extended Duration Substrate (34075, Pierce Biotechnology, Rockford, Illinois, United States). Intensities of corresponding protein bands were measured using an LAS-3000 mini image analyzer (Fuji Film, Japan), and the data within the linear detection range among the serial dilution of the samples were used.

If the endogenous Cdc protein was detected (we classified the antibody and the detected protein as Class I), the Cdc protein overexpression was quantified as the fold increase of the target protein over the endogenous protein amount (i.e., vector alone), which was normalized using hexokinase as a standard of total protein amount. The calculations are: Cdc overexpression in uracil− = *(Cdc_target_ura_* / *Cdc_vec_ura_)* / *(Hxk_target_ura_* / *Hxk_vec_ura_),* Cdc overexpression in leucine− = *(Cdc_target_leu_* / *Cdc_vec_ura_)* / *(Hxk_target_leu_* / *Hxk_vec_ura_),* where *Cdc* and *Hxk* mean the intensity of the target protein and hexokinase bands in the Western blot of the samples from indicated plasmid and culture conditions described in subscript (i.e., *target;* pTOW-target, *vec:* vector, *ura:* uracil− condition, *leu:* leucine− condition). If the endogenous Cdc protein was not detected, but the protein overexpressed from the pTOW-*target* was detected (we classified the antibody and the detected protein as Class II), we estimated the least-fold increase of the target protein using the number of the highest dilution in which the Cdc protein was detectable which was normalized using hexokinase. The calculations are: Least Cdc protein overexpression in uracil− = *MD* / *(Hxk_target_ura_* / *Hxk_vec_ura_),* Least Cdc protein overexpression in leu2− = *MD* × *(Cdc_target_leu_* / *Cdc_target_ura_)* / *(Hxk_target_leu_* / *Hxk_vec_ura_),* where *MD* is the maximum dilution number of the sample from the *target_ura* condition, in which the target Cdc protein band in Western blot could be detectable. At least two independent experiments were done for each target protein, and representative data were shown.

### Computation.

Computational prediction that systematically searches upper limit parameter values in the cell-cycle model has been described [19]. We used the Matlab code of the model with an algorithm that surveys and records the maximum fold increase of a certain parameter to maintain the normal cell cycle up to 256-fold [19]. To emulate the increase of the copy number of a target gene, we used the following way: If a target gene has only a single parameter for the gene expression, we increased it. If a target gene has two or more parameters for the gene expression, we increased them all together. If a target gene does not have a gene expression parameter but has the protein amount itself, then we simply increased the amount. Parameters tested are shown in Table S4. Some gene groups described in the model are listed as one gene because of redundant function, i.e., *CLB1*/*CLB2, CLB5*/*CLB6, CLN1*/*CLN2,* and *MBP1*/*SWI4*/*SWI6;* whereas we analyzed these groups as independent genes (Table S4). Computer simulations were done using Matlab version 7.0.4. The Matlab codes are provided in Text S1–S3.

## Supporting Information

Figure S1 A Possible Mechanism for the Linearity of the Growth Rate Copy Number Correlation in the gTOW Experiment (41 KB PDF)

Figure S2 A Scatter Plot between the Plasmid Copy Number in Uracil and Leucine Conditions in the gTOW Experiment (16 KB PDF)

Figure S3 A Scatter Plot between Protein Abundance and the Plasmid Copy Number in the gTOW Experiment (12 KB PDF)

Figure S4 Molecular Interaction Map of *CDC* Genes (181 KB PDF)

Figure S5 Copy Number of the Plasmid with Paralogous Gene Pairs Determined in the gTOW Experiment (13 KB PDF)

Figure S6 Quantification of the Protein Level Expressed in the gTOW Experiment (68 KB PDF)

Table S1 Doubling Time Determined in *gTOW* Experiments (19 KB PDF)

Table S2 Plasmid Copy Number Determined in *gTOW* Experiments (18 KB PDF)

Table S3 Doubling Time of Yeast Strains with *GAL1-CDCgene* Plasmids (8 KB PDF)

Table S4 Parameters Increased In Silico Upper Limit Gene Dosage Search (8 KB PDF)

Table S5 Quantification of Proteins Overexpressed in the gTOW (7 KB PDF)

Table S6 Primers Used to Construct Plasmids Containing Cell-Cycle Related Genes (17 KB PDF)

Text S1 Instruction for the Matlab Scripts (27 KB DOC)

Text S2 Matlab Script (1/2) for Parameter Analysis (8 KB DOC)

Text S3 Matlab Script (2/2) for Parameter Analysis (32 KB DOC)

## Abbreviations

CDK: cyclin-dependent kinase
gTOW: genetic tug-of-war
ORF: open reading frame
pTOW-*target*: plasmid for genetic tug-of-war
