# Correction: In Vivo Robustness Analysis of Cell Division Cycle in *Saccharomyces cerevisiae*

**DOI:** 10.1371/journal.pgen.0020218

**Published:** 2006-12-29

**Authors:** Hisao Moriya, Yuki Shimizu-Yoshida, Hiroaki Kitano

In *PLoS Genetics,* volume 2, issue 7: 10.1371/journal.pgen.0020111

In the Results section, the reference to Figure S7C and S7D is incorrect; the correct reference is to Figure S6D.

