# Supplementary material for: In Vivo Robustness Analysis of Cell Division Cycle Genes in Saccharomyces cerevisiae
Source: PLoS Genet. 2006 Jul 14;2(7):e111. doi: 10.1371/journal.pgen.0020111 (PMC1500812; doi:10.1371/journal.pgen.0020111)
Supplement: Supplementary file 1 [file pgen.0020111.sg001.pdf]

Moriya_Figure S1

A. In uracil- condition

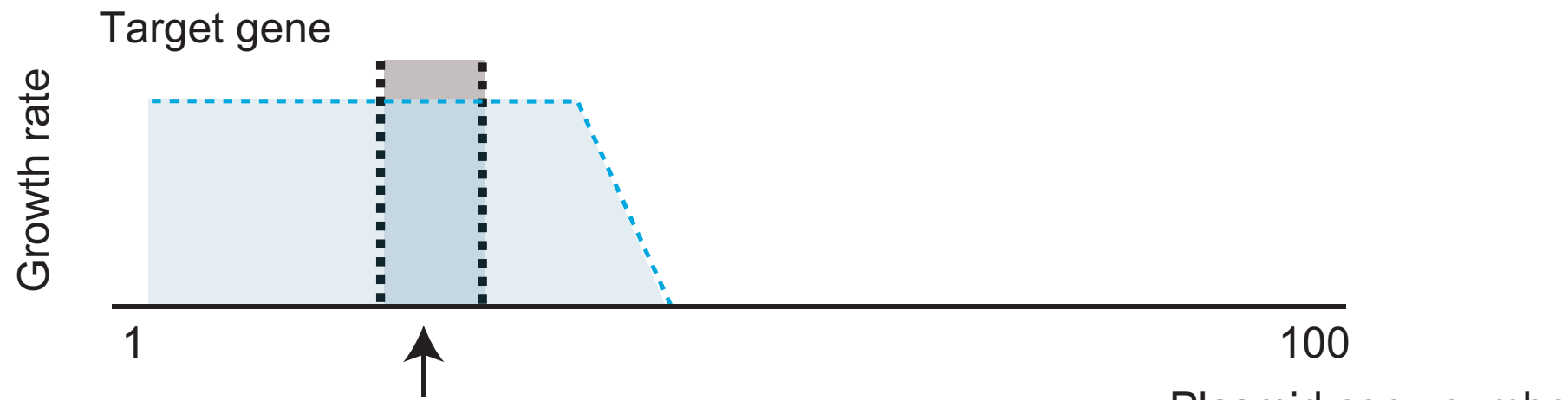

Plasmid copy number in uracil- condition is determined by the nature of 2μm plasmid, if the number is not above the upper limit of the target gene. This number is around 40 with a large variation.

B. In leucine- condition

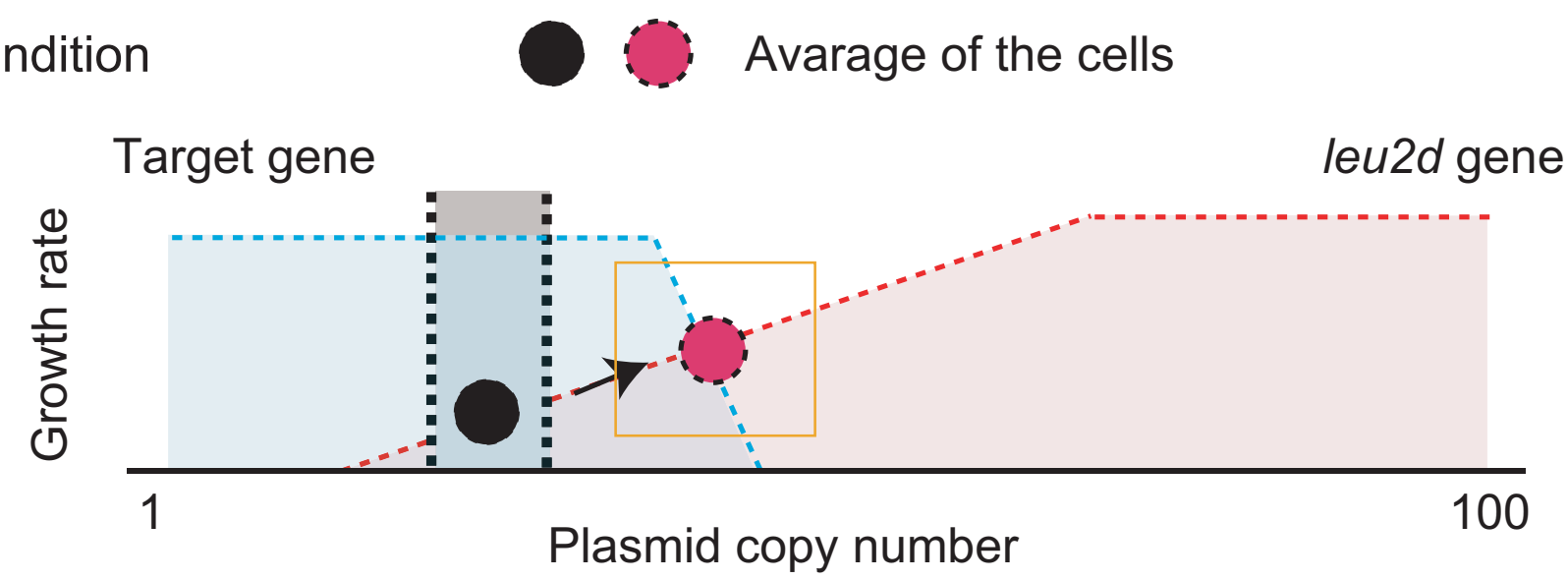

When the cells were transferred into leucine- condition, the cells have low average growth rate (black circle) which is determined by the linear copy number-fitness (growth rate) correlation of *leu2d* (pink dotted line). Then the average starts to increase (arrowhead) because the cells with higher plasmid copy number grow faster. This effect could be considered as a positive feedback loop between the plasmid copy number and the fitness. After certain generations of the cutivation, the average is determined in the point with the highest fitness, which is determined by the counter balance of fitness between *leu2d* and the target gene. The negative fitness effect could be considered as a negative feedback loop between plasmid copy number and the fitness.

C. In Orange area in B

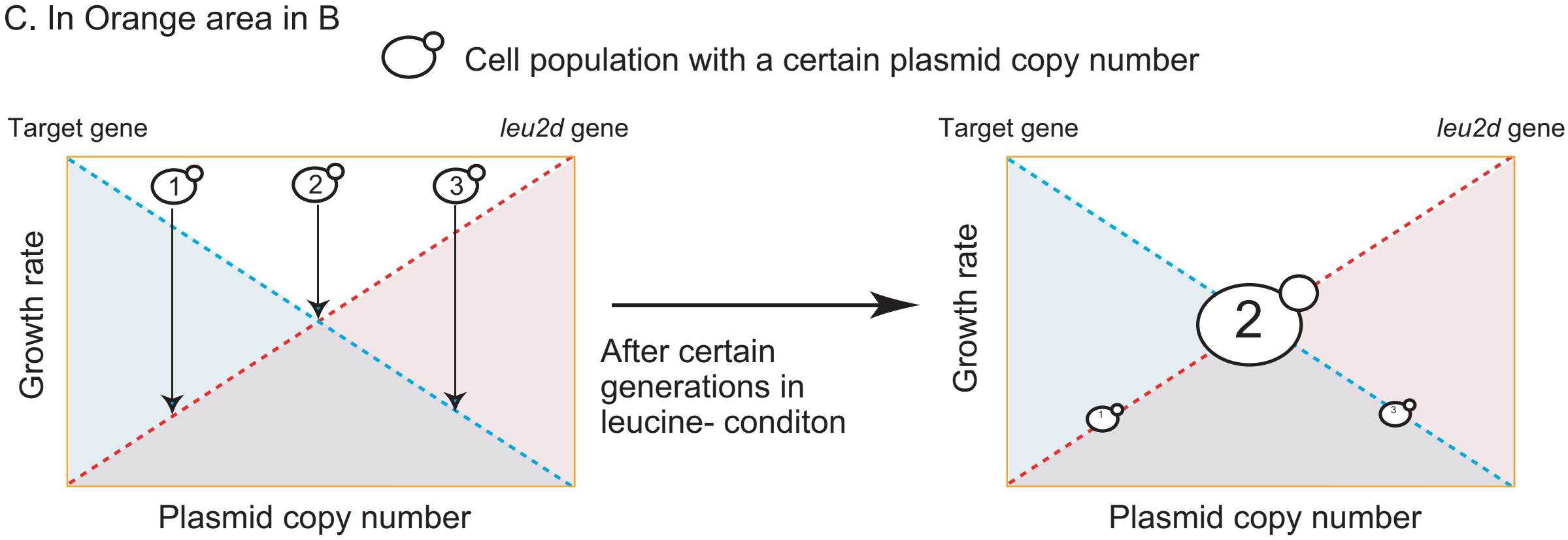

Orange area in B is shown. The growth rates of cells with different gTOW plasmid copy number (1, 2 and 3) should be determined by the limiting factor from either of *leu2d* or *CDC* gene. After certain generations in leucine- condition, the cell population with the highest fitness dominates the culture, which should be on the crossing point of the both lines.
