# Supplementary material for: In Vivo Robustness Analysis of Cell Division Cycle Genes in Saccharomyces cerevisiae
Source: PLoS Genet. 2006 Jul 14;2(7):e111. doi: 10.1371/journal.pgen.0020111 (PMC1500812; doi:10.1371/journal.pgen.0020111)
Supplement: Supplementary file 7 [file pgen.0020111.st001.pdf]

Table S1. Doubling time determined in *gTOW* experiments

	Genes	Leucine conc. (μg/ml)	Mean doubling time determined in *gTOW* experiments, min ± SD (fold growth inhibiton over vector alone)										
			Wild type									Frame shift	
			A		B	C	D	E	F	G		H	I
			0		20	25	40	50	60	100		0	100
1	*BUB2*		275 ± 15	(0.9)						225 ± 22	(1.1)	358 ± 20	218 ± 4
2	*CDC6*		333 ± 8	(1.1)						220 ± 4	(1.1)	372 ± 21	
3	*CDC14*		3350 ± 1070	(10.6)	3903 ± 988		2731 ± 973		659 ± 103	728 ± 79	(3.6)	387 ± 28	243 ± 15
4	*CDC15*		290 ± 16	(0.9)						224 ± 7	(1.1)	313 ± 19	224 ± 0
5	*CDC20*		496 ± 35	(1.6)						254 ± 20	(1.2)	428 ± 70	
6	*CDC28*		289 ± 15	(0.9)						216 ± 9	(1.1)	288 ± 22	229 ± 20
7	*CDH1*		325 ± 14	(1.0)						220 ± 18	(1.1)	288 ± 10	
8	*CLB1*		626 ± 26	(2.0)		370 ± 22		190 ± 5		219 ± 9	(1.1)	394 ± 56	235 ± 12
9	*CLB2*		2074 ± 142	(6.6)	1018 ± 147	677 ± 86	261 ± 16	216 ± 17	222 ± 16	240 ± 17	(1.2)	420 ± 25	246 ± 9
10	*CLB3*		4778 ± 936	(15.1)	6732 ± 2505		1923 ± 970	381 ± 56	271 ± 13	277 ± 16	(1.4)	289 ± 9	235 ± 18
11	*CLB4*		371 ± 20	(1.2)						255 ± 18	(1.3)	260 ± 28	215 ± 12
12	*CLB5*		3251 ± 904	(10.3)	1695 ± 166	1130 ± 194	284 ± 18	218 ± 8	206 ± 16	236 ± 23	(1.2)	430 ± 24	240 ± 13
13	*CLB6*		397 ± 38	(1.3)						275 ± 20	(1.4)	370 ± 37	
14	*CLN1*		381 ± 29	(1.2)						269 ± 11	(1.3)	366 ± 41	
15	*CLN2*		312 ± 14	(1.0)						245 ± 11	(1.2)	391 ± 39	
16	*CLN3*		318 ± 7	(1.0)						279 ± 23	(1.4)	355 ± 17	
17	*ESP1*		395 ± 19	(1.2)						257 ± 15	(1.3)	361 ± 44	
18	*LTE1*		367 ± 14	(1.2)						246 ± 11	(1.2)	370 ± 18	
19	*MAD2*		296 ± 15	(0.9)						239 ± 45	(1.2)	432 ± 64	
20	*MBP1*		346 ± 21	(1.1)						228 ± 14	(1.1)	364 ± 35	
21	*MCM1*		4381 ± 1404	(13.9)	1410 ± 407	1170 ± 267	302 ± 22	221 ± 7	230 ± 61	240 ± 20	(1.2)	431 ± 33	239 ± 15
22	*MIH1*		274 ± 17	(0.9)						239 ± 9	(1.2)	327 ± 25	226 ± 8
23	*NET1*		1173 ± 340	(3.7)	653 ± 46		244 ± 18	204 ± 5	212 ± 24	222 ± 15	(1.1)	335 ± 24	253 ± 22
24	*PDS1*		506 ± 66	(1.6)		285 ± 38		202 ± 19		200 ± 6	(1.0)	253 ± 17	237 ± 15
25	*SIC1*		2392 ± 145	(7.6)	768 ± 96		253 ± 14		212 ± 26	247 ± 12	(1.2)	607 ± 81	248 ± 21
26	*SWE1*		2336 ± 898	(7.4)	770 ± 137		250 ± 11		209 ± 17	262 ± 26	(1.3)	387 ± 37	262 ± 16
27	*SWI4*		404 ± 28	(1.3)						237 ± 18	(1.2)	333 ± 24	241 ± 16
28	*SWI5*		570 ± 70	(1.8)	409 ± 63		325 ± 30		262 ± 11	262 ± 21	(1.3)	358 ± 32	249 ± 19
29	*SWI6*		264 ± 24	(0.8)						201 ± 8	(1.0)	331 ± 27	
30	*TEM1*		259 ± 19	(0.8)						201 ± 5	(1.0)	389 ± 18	
31	vector (pSBI101)		316 ± 49	(1.0)	261 ± 51		204 ± 22		200 ± 18	204 ± 6	(1.0)		
