# Supplementary material for: In Vivo Robustness Analysis of Cell Division Cycle Genes in Saccharomyces cerevisiae
Source: PLoS Genet. 2006 Jul 14;2(7):e111. doi: 10.1371/journal.pgen.0020111 (PMC1500812; doi:10.1371/journal.pgen.0020111)
Supplement: Supplementary file 8 [file pgen.0020111.st002.pdf]

Table S2. Plasmid copy number determined in *gTOW* experiments

			Mean copy number per haploid genome, copy number ± SD								
			Wild type							Frame shift	
	Genes	Leucinc conc (μg/ml)	A<br>0	B<br>20	C<br>25	D<br>40	E<br>50	F<br>60	G<br>100	H<br>0	I<br>100
1	*BUB2*		127.9 ± 24.1						58.0 ± 7.5	143.7 ± 30.4	34.1 ± 14.9
2	*CDC6*		78.1 ± 17.2						23.7 ± 15.6	225.0 ± 24.0	41.5 ± 0.8
3	*CDC14*		0.8 ± 0.1	0.8 ± 0.1		1.2 ± 0.1		0.7 ± 0.1	0.8 ± 0.1	81.3 ± 10.1	26.5 ± 9.9
4	*CDC15*		166.6 ± 13.4						66.6 ± 8.4	217.5 ± 28.1	60.5 ± 20.4
5	*CDC20*		69.6 ± 4.6						19.0 ± 5.7	147.9 ± 5.7	24.9 ± 5.6
6	*CDC28*		83.7 ± 14.7						38.6 ± 4.1	157.5 ± 17.0	47.5 ± 5.9
7	*CDH1*		62.1 ± 1.4						17.7 ± 8.1	142.9 ± 18.5	92.0 ± 34.1
8	*CLB1*		71.1 ± 4.2		41.3 ± 5.6		17.2 ± 1.9		18.3 ± 4.1	199.4 ± 42.9	29.6 ± 2.5
9	*CLB2*			30.3 ± 3.7	19.0 ± 3.6	11.9 ± 0.7	7.8 ± 2.0	9.6 ± 1.3	8.1 ± 1.1	192.1 ± 18.1	29.0 ± 4.6
10	*CLB3*					17.9 ± 3.8	8.3 ± 1.0	7.7 ± 1.3	8.4 ± 1.8	176.6 ± 11.4	56.5 ± 5.2
11	*CLB4*		104.8 ± 12.5						45.9 ± 6.0	122.7 ± 26.2	55.5 ± 8.8
12	*CLB5*		12.8 ± 5.0		7.8 ± 0.8	4.4 ± 1.6	3.5 ± 0.8	5.3 ± 0.5	4.0 ± 0.8	361.2 ± 68.0	30.9 ± 3.9
13	*CLB6*		141.2 ± 23.8						37.8 ± 11.7	168.9 ± 32.8	41.0 ± 16.5
14	*CLN1*		55.7 ± 7.7						11.2 ± 5.0	175.6 ± 11.2	32.9 ± 14.5
15	*CLN2*		115.9 ± 21.1						42.8 ± 11.9	180.0 ± 28.4	37.4 ± 10.3
16	*CLN3*		73.6 ± 11.5						30.8 ± 7.2	171.5 ± 19.9	29.6 ± 6.4
17	*ESP1*		163.7 ± 6.9						40.0 ± 4.2	247.7 ± 28.9	47.1 ± 16.2
18	*LTE1*		125.4 ± 36.3						27.1 ± 9.6	201.3 ± 37.6	38.7 ± 6.0
19	*MAD2*		94.7 ± 13.1						33.7 ± 9.4	128.0 ± 14.6	23.0 ± 9.0
20	*MBP1*		94.6 ± 27.5						29.3 ± 5.8	185.6 ± 77.7	44.9 ± 25.2
21	*MCM1*		11.9 ± 4.2	10.3 ± 1.2	7.5 ± 1.5	2.8 ± 0.9	2.9 ± 0.8	2.0 ± 0.5	3.4 ± 0.7	131.4 ± 20.6	18.8 ± 0.4
22	*MIH1*		197.0 ± 30.7						90.7 ± 12.9	184.1 ± 13.3	31.7 ± 4.4
23	*NET1*		49.9 ± 4.3	38.6 ± 5.6		15.6 ± 2.0	11.5 ± 3.5	14.6 ± 2.2	12.2 ± 2.5	180.6 ± 27.4	58.2 ± 3.9
24	*PDS1*		55.8 ± 3.2		49.8 ± 7.5		21.3 ± 3.3		21.3 ± 1.9	190.7 ± 18.6	71.0 ± 7.0
25	*SIC1*		30.9 ± 4.3	31.3 ± 1.6		13.6 ± 0.4		10.9 ± 0.7	10.1 ± 1.8	233.8 ± 41.9	30.1 ± 1.7
26	*SWE1*		22.8 ± 2.0	17.8 ± 1.8		9.7 ± 2.3		8.4 ± 1.2	7.2 ± 2.2	230.9 ± 49.0	40.9 ± 6.6
27	*SWI4*		56.9 ± 7.5						17.4 ± 3.9	253.4 ± 5.5	71.3 ± 3.7
28	*SWI5*		61.5 ± 5.2	64.0 ± 10.5		28.5 ± 5.0		15.7 ± 6.1	15.9 ± 5.1	102.6 ± 14.3	46.4 ± 5.5
29	*SWI6*		154.3 ± 18.0						48.0 ± 16.0	124.6 ± 4.4	28.8 ± 6.0
30	*TEM1*		86.7 ± 22.6						34.6 ± 13.6	133.6 ± 7.0	27.9 ± 7.0
31	vector (pSBI40		162.6 ± 39.5	83.0 ± 13.5		62.1 ± 22.5		56.3 ± 12.3	32.8 ± 14.2		
32	WT		1.2 ± 0.1						1.0 ± 0.2		
