# Supplementary material for: In Vivo Robustness Analysis of Cell Division Cycle Genes in Saccharomyces cerevisiae
Source: PLoS Genet. 2006 Jul 14;2(7):e111. doi: 10.1371/journal.pgen.0020111 (PMC1500812; doi:10.1371/journal.pgen.0020111)
Supplement: Supplementary file 9 [file pgen.0020111.st003.pdf]

Table S3. Doubling time of yeast strins with *GAL1-CDCgene* plasmids

		Mean doubling time, min ± SD (fold delay over vector alone)					
	genes	YM -uracil +2% galactose			YM -leucine +2% glucose		
1	*BUB2*	409	± 35	(1.1)	270	± 3	(0.9)
2	*CDC6*	330	± 29	(0.9)	287	± 8	(1.0)
3	*CDC14*	3013	± 217	(8.0)	282	± 15	(1.0)
4	*CDC15*	404	± 34	(1.1)	311	± 15	(1.1)
5	*CDC20*	1330	± 724	(3.5)	294	± 15	(1.0)
6	*CDC28*	345	± 26	(0.9)	320	± 11	(1.1)
7	*CDH1*	476	± 24	(1.3)	274	± 20	(1.0)
8	*CLB1*	1097	± 128	(2.9)	301	± 19	(1.0)
9	*CLB2*	1949	± 531	(5.2)	299	± 17	(1.0)
10	*CLB3*	1057	± 205	(2.8)	337	± 36	(1.2)
11	*CLB4*	672	± 76	(1.8)	328	± 31	(1.1)
12	*CLB5*	999	± 88	(2.6)	317	± 24	(1.1)
13	*CLB6*	1796	± 141	(4.8)	303	± 30	(1.1)
14	*CLN1*	346	± 13	(0.9)	294	± 27	(1.0)
15	*CLN2*	495	± 145	(1.3)	320	± 23	(1.1)
16	*CLN3*	426	± 25	(1.1)	311	± 15	(1.1)
17	*ESP1*	297	± 15	(0.8)	299	± 26	(1.0)
18	*LTE1*	527	± 19	(1.4)	352	± 15	(1.2)
19	*MAD2*	728	± 78	(1.9)	264	± 21	(0.9)
20	*MBP1*	785	± 28	(2.1)	334	± 21	(1.2)
21	*MCM1*	5588	± 1403	(14.8)	329	± 25	(1.1)
22	*MIH1*	449	± 24	(1.2)	295	± 21	(1.0)
23	*NET1*	4338	± 655	(11.5)	351	± 45	(1.2)
24	*PDS1*	4232	± 1211	(11.2)	285	± 22	(1.0)
25	*SIC1*	1773	± 506	(4.7)	471	± 26	(1.6)
26	*SWE1*	2393	± 573	(6.3)	338	± 35	(1.2)
27	*SWI4*	280	± 14	(0.7)	405	± 23	(1.4)
28	*SWI5*	1417	± 290	(3.7)	302	± 23	(1.0)
29	*SWI6*	408	± 36	(1.1)	295	± 19	(1.0)
30	*TEM1*	445	± 20	(1.2)	259	± 22	(0.9)
31	vector (pSBI104)	378	± 21	(1.0)	288	± 5	(1.0)
