# Supplementary material for: In Vivo Robustness Analysis of Cell Division Cycle Genes in Saccharomyces cerevisiae
Source: PLoS Genet. 2006 Jul 14;2(7):e111. doi: 10.1371/journal.pgen.0020111 (PMC1500812; doi:10.1371/journal.pgen.0020111)
Supplement: Supplementary file 10 [file pgen.0020111.st004.pdf]

Table S4. Parameters increased *in silico* upper limit gene dosage search

Gene	gTOW *in vivo* #	Max up *in silico*	parameter category	Parameters changed Parameter1	Parameter2	Parameter	Parameter4
BUB2	118.5	32.0	constant	Bub2l	Bub2h		
CDC6	79.1	2.8	synthesis (constant, 2 regulated)	ksf6_r	ksf6_c	ksf6_r2	
CDC14	1.8	2.0	synthesis (constant)	ks14			
CDC15	157.7	256.0	constant	Cdc15T			
CDC20	70.6	16.0	synthesis (constant, regulated)	ks20_r	ks20_c		
CDH1	63.1	90.5	synthesis (constant)	kscdh			
*CLB1**	72.1	8.0	synthesis (constant, regulted)	ksb2_r	ksb2_c		
*CLB2**	31.3	8.0	synthesis (constant, regulted)	ksb2_r	ksb2_c		
*CLB5**	8.8	32.0	synthesis (constant, regulated)	ksb5_c	ksb5_r		
*CLB6**	142.2	32.0	synthesis (constant, regulated)	ksb5_c	ksb5_r		
*CLN1**	56.7	5.7	synthesis (regulated)	ksn2_r			
*CLN2**	116.9	5.7	synthesis (regulated)	ksn2_r			
CLN3	74.6	16.0	synthesis (assoc.MASS)	C0			
ESP1	164.7	1.4	constant	ESP1T			
LTE1	126.4	256.0	constant	lte1l	lte1h		
MAD2	95.7	128.0	constant	ki20_c	ki20_c		
*MBP1**	95.6	4.0	GK function	ksn2_r	ksb5_r	ksf6_r2	ks1pds_r
MCM1	11.3	16.0	GK function	ks2pds_r	ksswi_r	ksb2_r	ks20_r
NET1	39.6	2.0	synthesis (constant)	ksnet			
PDS1	73.9	1.4	synthesis (2 regulated)	ks2pds_r	ks1pds_r		
SIC1	32.3	2.8	synthesis (constant, regulated)	ksc1_r	ksc1_c		
*SWI4**	54.5	4.0	GK function	ksn2_r	ksb5_r	ksf6_r2	ks1pds_r
SWI5	62.5	2.0	synthesis (constant, regulated)	ksswi_r	ksswi_c		
*SWI6**	155.3	4.0	GK function	ksn2_r	ksb5_r	ksf6_r2	ks1pds_r
TEM1	87.7	256.0	constant	TEM1T			

* *CLB1* /*CLB2* , *CLB5* /*CLB6* , *CLN1* /*CLN2* and *MBP1* /*SWI4* /*SWI6* are implemented as single genes in the computational model. # The number is the copy number determined in the gTOW experiment (plasmid copy) + 1 (chromosomal copy).
