# Supplementary material for: In Vivo Robustness Analysis of Cell Division Cycle Genes in Saccharomyces cerevisiae
Source: PLoS Genet. 2006 Jul 14;2(7):e111. doi: 10.1371/journal.pgen.0020111 (PMC1500812; doi:10.1371/journal.pgen.0020111)
Supplement: Supplementary file 11 [file pgen.0020111.st005.pdf]

Table S5. Quantification of proteins over-expressed in the gTOW

No.	Gene Name	Class*1	Gene copy number		Protein level (fold	
			uracil–	leucin– *3	uracil–	leucine– *3
2	*CDC6*	I	18.63	49.17	38.36	60.73
3	*CDC14*	I		2.48		1.75
4	*CDC15*	I	82.57	165.28	73.11	235.37
5	*CDC20*	I	20.20	49.80	10.29	48.63
6	*CDC28*	II	47.53	102.13	> 32	> 80
7	*CDH1*	I	22.56	82.01	31.55	65.53
8	*CLB1*	I	36.26	93.41	20.47	88.46
9	*CLB2*	I	9.88	14.00	9.81	12.35
10	*CLB3*	I	16.78	13.55	19.20	16.00
12	*CLB5*	I	7.87	11.06	10.94	10.21
13	*CLB6*	II	30.24	180.77	> 8	> 17
15	*CLN2*	II	28.10	148.03	> 2	> 33
16	*CLN3*	II	32.56	82.01	> 4	> 12
18	*LTE1*	II	22.71	160.79	> 4	> 145
19	*MAD2*	I	38.75	107.15	40.30	93.87
23	*NET1*	II	24.10	37.00	> 16	> 34
26	*SWE1*	I	9.90	12.30	11.39	10.34
29	*SWI6*	I	40.90	149.10	3.57	8.48
30	*TEM1*	II	61.55	118.78	> 16	> 103

*1 I: The endogenous protein was detected, II: Only over-expressed protein was detected. *2 The number is the copy number determined in the gTOW experiment (plasmid copy) + 1 (chromosomal copy). *3 20 mg/ml (*CLB2* , *CLB5* and *SWE1*), 30 mg/ml (*CLB3*) and 100 mg/ml (*CDC14*) leucine were added in the growth medium.
