# Supplementary material for: In Vivo Robustness Analysis of Cell Division Cycle Genes in Saccharomyces cerevisiae
Source: PLoS Genet. 2006 Jul 14;2(7):e111. doi: 10.1371/journal.pgen.0020111 (PMC1500812; doi:10.1371/journal.pgen.0020111)
Supplement: Supplementary file 12 [file pgen.0020111.st006.pdf]

Table S6A. Primers used to construct plasmids containing cell cycle related genes

No.	ORF Name	Gene Name	Up primer (from just downstream of upstream ORF)		Down primer (from just upstream of downstream ORF)		Frame shift forward primer		Frame shift reverse primer		*GAL* primer	
1	*YMR055C*	*BUB2*	OSBI0139	agctatgaccatgattacgccaagcttTGCAACTCATAATGTTTGGC	OSBI0140	tagctagttttactacatcgcggtaccGAGCCATTTTCATTTCGTGC	OSBI0278	acttgttaacttttgcATG**a**ACCTCAATTGAAGATCTG	OSBI0279	GAGGT**t**CATgcaaaagttaacaagtttaaaagtctg	OSBI0249	ctttaacgtcaaggagaaaaaactata**ATG**ACCTCAATTGAAGATCTG
2	*YJL194W*	*CDC6*	OSBI0042	agctatgaccatgattacgccaagcttTACTTGCTTGCAAATTTAGC	OSBI0043	tagctagttttactacatcgcggtaccTCGTTTTCCCCTTTTTCTTC	OSBI0329	caatagtaccatatacgttcATG**a**TCAGCTATACCAATAACTCC	OSBI0330	GGAGTTATTGGTATAGCTGA**t**CATgaacgtatatggtactattg	OSBI0250	ctttaacgtcaaggagaaaaaactata**ATG**TCAGCTATACCAATAACTCC
3	*YFR028C*	*CDC14*	OSBI0505	agctatgaccatgattacgccaagctCCCCACTTTTGTCTATGAGAGTGCAT	OSBI0045	tagctagttttactacatcgcggtaccACTCTTTCGAAAAGACAGCG	OSBI0280	GCGTAGGAGTGTATACCTCG**a**ACAACACGATCGAGTTCCTG	OSBI0281	CAGGAACTCGATCGTGTTGT**t**CGAGGTATACACTCCTACGC	OSBI0251	ctttaacgtcaaggagaaaaaactata**ATG**CGTAGGAGTGTATACCTCG
4	*YAR019C*	*CDC15*	OSBI0141	agctatgaccatgattacgccaagcttctaAGCTGCTCACCCAACTG	OSBI0142	tagctagttttactacatcgcggtaccCTATGcatACTCACACAGAG	OSBI0282	gactataccATG**a**AACAGTATGGCCGATACCG	OSBI0283	GGCCATACTGTT**t**CATggtatagtcttaccagg	OSBI0252	ctttaacgtcaaggagaaaaaactata**ATG**AACAGTATGGCCGATACCG
5	*YGL116W*	*CDC20*	OSBI0054	agctatgaccatgattacgccaagcATAATTTGACATTCATAATA	OSBI0055	tagctagttttactacatcgcggtaccTTATGTATTTTCCTTTTCGC	OSBI0331	gacaagtattacaaagaagactaATG**a**CCAGAAAGCTCTAGAGATAAG	OSBI0332	CTTATCTCTAGAGCTTTCTGG**t**CATtagtcttctttgtaatacttgtc	OSBI0253	ctttaacgtcaaggagaaaaaactata**ATG**CCAGAAAGCTCTAGAG
6	*YBR160W*	*CDC28*	OSBI0013	agctatgaccatgattacgccaagcttTTATCGTTCTCGAGATAG	OSBI0014	ttactacatcgcggtaccATGAATAGCACGTATTAATTGGTTTAC	OSBI0030	ccttcgaacATG**a**AGCGGTGAATTAGCAAATTAC	OSBI0031	TAATTCACCGCT**t**CATgttcgaaggatagttc	OSBI0254	ctttaacgtcaaggagaaaaaactata**ATG**AGCGGTGAATTAGC
7	*YGL003C*	*CDH1*	OSBI0028	agctatgaccatgattacgccaagcttTTGGCAAATCTAGATCC	OSBI0029	tagctagttttactacatcgcggtaccTTGTCGTTTCTATTGTAC					OSBI0255	ctttaacgtcaaggagaaaaaactata**ATG**TCCACAAACCTGAACCC
8	*YGR108W*	*CLB1*	OSBI0046	agctatgaccatgattacgccaagcttATTAATCATTTTCAACTTGC	OSBI0047	tagctagttttactacatcgcggtaccAACATAGATCAAAATTATAG	OSBI0165	ggaagctttaatcttctcataATG**a**TCACGATCCCTTTTGGTAGAG	OSBI0166	CTCTACCAAAAGGGATCGTGA**t**CATtatgagaagattaaagcttcc	OSBI0256	ctttaacgtcaaggagaaaaaactata**ATG**TCACGATCCCTTTTGGTAG
9	*YPR119W*	*CLB2*	OSBI0048	agctatgaccatgattacgccaagcttGTGGTTTTGACTAAACAGCC	OSBI0049	tagctagttttactacatcgcggtaccGATTAGTACTAGTAATGCTC	OSBI0146	cttcattgatcttatagATG**a**TCCAACCCAATAGAAAACAC	OSBI0147	GTGTTTTCTATTGGGTTGGA**t**CATctataagatcaatgaag	OSBI0257	ctttaacgtcaaggagaaaaaactata**ATG**TCCAACCCAATAGAAAACAC
10	*YDL155W*	*CLB3*	OSBI0290	agctatgaccatgattacgccaagcttAAAACATCTCAGTTACCCGC	OSBI0291	tagctagttttactacatcgcggtacCATATTCCATTTGGTGGCAG	OSBI0302	cattgcttattataaATG**a**CATCATAACTCACAGTCTTTG	OSBI0303	CAAAGACTGTGAGTTATGATG**t**CATttataataagcaatg	OSBI0298	ctttaacgtcaaggagaaaaaactata**ATG**CATCATAACTCACAGTC
11	*YLR210W*	*CLB4*	OSBI0292	agctatgaccatgattacgccaagctTCTTTTAAGTTTATTCGGAG	OSBI0293	tagctagttttactacatcgcggtaccGAAGCAAAGAAGGGATAAGC	OSBI0304	caaacaaggaaattgacagATG**a**ATGCTTGAAGGGTATACGG	OSBI0305	CCGTATACCCTTCAAGCAT**t**CATctgtcaatttccttgtttg	OSBI0299	ctttaacgtcaaggagaaaaaactata**ATG**ATGCTTGAAGGGTATAC
12	*YPR120C*	*CLB5*	OSBI0143	agctatgaccatgattacgccaagcttGTTCGATCCTGAGTGCGAGC	OSBI0144	tagctagttttactacatcgcggtaccATGAGAAGAGGGGCAGATGC	OSBI0167	gccgctgaacacctttactgaacaATG**a**GGAGAGAACCACGACCATGAGC	OSBI0168	GCTCATGGTCGTGGTTCTCTCC**t**CATtgttcagtaaaggtgttcagcggc	OSBI0258	ctttaacgtcaaggagaaaaaactata**ATG**GGAGAGAACCACGACCATG
13	*YGR109C*	*CLB6*	OSBI0052	agctatgaccatgattacgccaagcttTTTTTATTAACTAGGTGGTC	OSBI0053	tagctagttttactacatcgcggtaccAAGGTGCTAGCCTACACAGA	OSBI0333	ctctccctcttttaaatttttaaaATG**a**AATTGTATCCCTAGTCCAATTAG	OSBI0334	CTAATTGGACTAGGGATACAATT**t**CATtttaaaaatttaaaaggagggagag	OSBI0259	ctttaacgtcaaggagaaaaaactata**ATG**AATTGTATCCCTAGTCC
14	*YMR199W*	*CLN1*	OSBI0036	agctatgaccatgattacgccaagcttAGAAGGCCAGATCTTGTGCG	OSBI0037	tagctagttttactacatcgcggtaccTAGTAATAAAGTGCAACAAA	OSBI0387	CATGAACCACTCAGAAGTGAAAACTG**a**GGTTAATTGTCACTGCAAAGCAG	OSBI0388	CTGCTTTGCAGTGACAATTAACC**t**CAGTTTTCACTTCTGAGTGGTTCATg	OSBI0260	ctttaacgtcaaggagaaaaaactata**ATG**AACCACTCAGAAGTGAAAAC
15	*YPL256C*	*CLN2*	OSBI0038	agctatgaccatgattacgccaagcttTTGTTCAGTAGTCATTCGCC	OSBI0039	tagctagttttactacatcgcggtaccATGGAATGGTAGTCGAAAGG	OSBI0337	cattcattaaatttaacgacagacaATG**a**GCTAGTGCTGAACCAAGACC	OSBI0338	GGTCTTGGTTCAGCACTAGC**t**CATtgtctgtcgttaaatttaatgaatg	OSBI0261	ctttaacgtcaaggagaaaaaactata**ATG**GCTAGTGCTGAACCAAGACC
16	*YAL040C*	*CLN3*	OSBI0040	agctatgaccatgattacgccaagcttAATGATATACAGCCAGCGTA	OSBI0041	tagctagttttactacatcgcggtaccATAAATAAAACTAGTATACA	OSBI0339	gatttgatacgctttctgtacgATG**a**GCCATATTGAAGGATACCATAATTAG	OSBI0340	CTAATTATGGTATCCTTCAATATGGC**t**CATcgtacagaaagcgtatcaaatc	OSBI0262	ctttaacgtcaaggagaaaaaactata**ATG**GCCATATTGAAGGATACC
17	*YGR098C*	*ESP1*	OSBI0083	agctatgaccatgattacgccaagcttTTTCATGAGGAATTAAATGC	OSBI0084	tagctagttttactacatcgcggtaccATTGTTACGCCTTAACTTTT	OSBI0341	gaagtgcttataaattaaggaatgATG**a**GTAAAACAGGAGGAACCGCTG	OSBI0342	CAGCGGTTCCTCCTGTTTTAC**t**CATcattccttaatttataagcacttc	OSBI0263	ctttaacgtcaaggagaaaaaactata**ATG**GTAAAACAGGAGGAACCGC
18	*YAL024C*	*LTE1*	OSBI0073	agctatgaccatgattacgccaagcttAATTAAGATCATCGTAGTAA	OSBI0074	tagctagttttactacatcgcggtaccTATCCTCGTTGTATTCTAAC	OSBI0343	gattcgctaccctgaaccATG**a**GAAATATTTAGCCAGAAAGATTACTAC	OSBI0344	GTAGTAATCTTTCTGGCTAAATATTTC**t**CATggttcagggtagcgaatc	OSBI0264	ctttaacgtcaaggagaaaaaactata**ATG**GAAATATTTAGCCAGAAAG
19	*YJL030W*	*MAD2*	OSBI0154	agctatgaccatgattacgccaagcttTACCATTGCTGACCTACTGC	OSBI0155	tagctagttttactacatcgcggtaccTGAAAAAACGCATGAAGTGG	OSBI0345	gcccaatagcacatttaATG**a**TCACAATCAATATCACTAAAGGG	OSBI0346	CCCTTTAGTGATATTGATTGTGA**t**CATtaaatgtgctattgggc	OSBI0265	ctttaacgtcaaggagaaaaaactata**ATG**TCACAATCAATATCAC
20	*YDL056W*	*MBP1*	OSBI0077	agctatgaccatgattacgccaagcttGACCATACAACTACATATTA	OSBI0078	tagctagttttactacatcgcggtaccAGTTAGGAAAACGAACTGTA	OSBI0129	gtaaatcccagaaacacaagcATG**a**TCTAACCAAATATACTCAGCG	OSBI0130	CGCTGAGTATATTTGGTTAGA**t**CATgcttgtgtttctgggatttac	OSBI0266	ctttaacgtcaaggagaaaaaactata**ATG**TCTAACCAAATATACTCAG
21	*YMR043W*	*MCM1*	OSBI0079	agctatgaccatgattacgccaagcttAACATGCGAGAGTAAGAGAT	OSBI0080	tagctagttttactacatcgcggtaccTTATCGGAGAATGACACGCC	OSBI0152	gccacccagcaaaaATG**a**TCAGACATCGAAGAAGGTAC	OSBI0153	GTACCTTCTTCGATGTCTGA**t**CATttttgctgggtggc	OSBI0267	ctttaacgtcaaggagaaaaaactata**ATG**TCAGACATCGAAGAAGGTAC
22	*YMR036C*	*MIH1*	OSBI0096	agctatgaccatgattacgccaagcttAATTCGATAGATCTTTCTTC	OSBI0097	tagctagttttactacatcgcggtaccGAATTTAGAAGTGACCCCGG	OSBI0244	gtcagcgagggtgaagaaaccATG**a**AACAATATATTTCATGGAACTG	OSBI0245	CAGTTCCATGAAATATATTGTT**t**CATggtttcttcaccctcgctgac	OSBI0268	ctttaacgtcaaggagaaaaaactata**ATG**AACAATATATTTCATGGAAC
23	*YJL076W*	*NET1*	OSBI0156	agctatgaccatgattacgccaagcttTTATGTTACCCGTTCCACATG	OSBI0157	tagctagttttactacatcgcggtaccAATTCGCTGAAGTCTAAGAG	OSBI0169	ctgtacaccgtgcctccttctATG**a**TACAAGCTACAAGTGGTCTTGGTGC	OSBI0170	GCACCAAGACCACTTGTAGCTTGTA**t**CATagaaggaggcacggtgtacag	OSBI0269	ctttaacgtcaaggagaaaaaactata**ATG**TACAAGCTACAAGTGGTC
24	*YDR113C*	*PDS1*	OSBI0081	agctatgaccatgattacgccaagcttTTCCGTCCTGCGATTACTAC	OSBI0082	tagctagttttactacatcgcggtaccTGTGTTCTTACTTCTTCTGT	OSBI0150	gtgctagataataaacctttATGATG**a**CCAGCTAACGAAGATAAAGAG	OSBI0151	CTCTTTATCTTCGTTAGCTGG**t**CATCATaaaggtttattatctagcac	OSBI0270	ctttaacgtcaaggagaaaaaactata**ATG**ATGCCAGCTAACGAAG
25	*YLR079W*	*SIC1*	OSBI0015	agctatgaccatgattacgccaagcttCCCCCTCAAATCAATATG	OSBI0016	ttactacatcgcggtaccATGAATAGCCTCCCCTAACTCGCTTTG	OSBI0131	gggactattacacgaaaATG**a**ACTCCTTCCACCCCACCAAG	OSBI0132	CTTGGTGGGGTGGAAGGAGT**t**CATtttcgtgtaatagtccc	OSBI0271	ctttaacgtcaaggagaaaaaactata**ATG**ACTCCTTCCACCCCACCAAG
26	*YJL187C*	*SWE1*	OSBI0098	agctatgaccatgattacgccaagcttCCTGAACTATTAGTATATCC	OSBI0099	tagctagttttactacatcgcggtaccCTTCCATCCTTCCCTTATTC	OSBI0133	ggcgcacacgagaacagATG**a**AGTTCTTTGGACGAGGATGAAG	OSBI0134	CTTCATCCTCGTCCAAAGAACT**t**CATctgttctcgtgtgcgcc	OSBI0272	ctttaacgtcaaggagaaaaaactata**ATG**AGTTCTTTGGACGAGGATG
27	*YER111C*	*SWI4*	OSBI0145	agctatgaccatgattacgccaagcttTGCATTTGTTGTCCCTTCGC	OSBI0103	tagctagttttactacatcgcggtaccAAGTGTTTTTCGTCCCTCAG	OSBI0284	cgagaaatcATG**a**CCATTTGATGTTTTGATATC	OSBI0285	AACATCAAATGG**t**CATgatttctcgaataaatcaacc	OSBI0273	ctttaacgtcaaggagaaaaaactata**ATG**CCATTTGATGTTTTGATATC
28	*YDR146C*	*SWI5*	OSBI0286	agctatgaccatgattacgccaagcttATGCCAAATTGCAGATCTG	OSBI0287	tagctagttttactacatcgcggtaccATTATGTTACCCACATTCTC	OSIB0295	tcgaaaaagATG**a**GATACATCAAACTCTTGG	OSBI0296	GTTTGATGTATC**t**CATctttttcgaaaaaacaatgacg	OSBI0294	ctttaacgtcaaggagaaaaaactata**ATG**TTAGCAGAGTTGGACGTAGC
29	*YLR182W*	*SWI6*	OSBI0089	agctatgaccatgattacgccaagcttTAGAATTTATTTAAAATGTG	OSBI0090	tagctagttttactacatcgcggtaccGAACATTTTATTCCATCGGA	OSBI0347	gtataattctcgagaggATG**a**GCGTTGGAAGAAGTGGTACG	OSBI0348	CGTACCACTTCTTCCAACGC**t**CATcctctcgagaattatac	OSBI0275	ctttaacgtcaaggagaaaaaactata**ATG**GCGTTGGAAGAAGTGGTACG
30	*YML064C*	*TEM1*	OSBI0091	agctatgaccatgattacgccaagcttTGATTAGTCTAGATCGGTTT	OSBI0092	tagctagttttactacatcgcggtaccGATTCGGTTTTTATTATTCA	OSBI0349	gtagaaattaaattgagttaagctaagATG**a**GCTACACCAAGCACGGGAGC	OSBI0350	GCTCCCGTGCTTGGTGTAGC**t**CATcttagcttaactcaatttaatttctac	OSBI0276	ctttaacgtcaaggagaaaaaactata**ATG**GCTACACCAAGCACGGGAGC

Up primer	Down primer	Frame shift forward primer	Frame shift reverse primer	*GAL* primer
lower case: vector sequence	lower case: vector sequence	lower case: upsetram sequence	lower case: upsetram sequence	lower case: vector (*GAL1* promoter) sequence
upper case: target gene sequence	upper case: target gene sequence	upper case: coding sequence	upper case: coding sequence	upper case: target gene sequence from ATG (ATG is shown in bold letter)
		bold letter: insertion	bold letter: insertion	

Table S6B. Primers used in kinetic PCRs

Primers to amplify *LEU2* locus (plasmid)		
LEU2-F	GCTAATGTTTTGGCCTCTTC	*LEU2* +577 to +596 from ATG
LEU2-R	ATTTAGGTGGGTTGGGTTCT	*LEU2* +717 to +698 from ATG
Primers to amplify *LEU3* locus (genome)		
LEU3-F	CAGCAACTAAGGACAAGG	*LEU3* +2125 to +2142 from ATG
LEU3-R	GGTCGTTAATGAGCTTCC	*LEU2* +2259 to +2242 from ATG

Table S6C. Primers used to construct frame shift mutations

OSBI159	aacagtaccaccgaagtcgg	sequence downstream of pSBI40 cloning site
OSBI160	acgacaggtttcccgactgg	sequence upstream of pSBI40 cloning site

Table S6D. Primers used to construct pSBI103 (*GAL* system)

OSBI0246	ATTGTGAGCGGATAACAATTTCACACAGGATCCggatggacgcaaagaag
OSBI0247	CCAAACCTCTGGCGAAGAAGTCCAAAGCTTcattatagtttttttctccttgacg

upper case: pSBI40 sequence
lower cae: *GAL1* promoter sequence
