# Supplementary material for: In Vivo Robustness Analysis of Cell Division Cycle Genes in Saccharomyces cerevisiae
Source: PLoS Genet. 2006 Jul 14;2(7):e111. doi: 10.1371/journal.pgen.0020111 (PMC1500812; doi:10.1371/journal.pgen.0020111)
Supplement: Supplementary file 13 [file pgen.0020111.sd001.doc]

These are MATLAB scripts to test upper limit gene copy numbers in the Matlab code of Chen's 2004 budding yeast cell cycle model. To run the parameter test using the codes below, you need the body code files that can be obtained from Dr. Fred R. Cross [1]. Put these files into the same folder of the body code files.

To run parameter test, first rename ‘text S2’　‘partest_morya_special.m’, and ‘text S2’　‘cell03_partest_moriya_sp.m’. Open ‘partest_moriya_special.m’ then change parameter name in line 5 to whatever you like (in the original file, ksn2; cln2 synthesis, is entered), and the positions of parameters in the matrix in ‘Parameter names.xls’ (in the original file, [2,1] corresponding to ksn2_c and [2,2] corresponding to ksn2_r, are entered) of the body code files. Change the parameter name in line 6 (in the original, ksn2) to the same in line 5. Then, save and run this m-file.

1. Chen KC, Calzone L, Csikasz-Nagy A, Cross FR, Novak B, et al. (2004) Integrative analysis of cell cycle control in budding yeast. Mol Biol Cell 15: 3841-3862.
