# Supplementary material for: In Vivo Robustness Analysis of Cell Division Cycle Genes in Saccharomyces cerevisiae
Source: PLoS Genet. 2006 Jul 14;2(7):e111. doi: 10.1371/journal.pgen.0020111 (PMC1500812; doi:10.1371/journal.pgen.0020111)
Supplement: Supplementary file 14 [file pgen.0020111.sd002.doc]

% RENAME THIS FILE 'partest_moriya_special.m'

% script partest_script
% Set default initial condition.
cell03_setup
params;
ksn2 = [2, 1; 2, 2];
wt_report=cell03_partest_moriya_sp(init,p_names,ksn2)
