# Supplementary material for: In Vivo Robustness Analysis of Cell Division Cycle Genes in Saccharomyces cerevisiae
Source: PLoS Genet. 2006 Jul 14;2(7):e111. doi: 10.1371/journal.pgen.0020111 (PMC1500812; doi:10.1371/journal.pgen.0020111)
Supplement: Supplementary file 15 [file pgen.0020111.sd003.doc]

% RENAME THIS FILE 'cell03_partest_moriya_sp.m'

function table=cell03_partest_moriya_sp(init, p_names, testd_parms)
 
%Single parameter variation, saving diagnostic information on how model
%failed at the minimum decrement or increment of a given parameter that
%killed it.  'init' are initial conditions, p_names is an array with the
%same size as params, with the names of the parameters.
 
global params;
 
std_pars=params;
[rows,cols]=size(params);
table=zeros(rows, 5*cols );
 
%Set maximum power of two tested and the fineness of the binary search
%(fraction of power of two tested).
max_power=8;
precision=0.5;
 
%Set the number of changed parameters.
[testd_rows, testd_cols]=size(testd_parms);
 
    %Do the test only for non-zero parameters!
        pass_exp=0;
        fail_exp=0;
        go_calculation=0;
 
        for row=1:testd_rows
            i=testd_parms(row,1);
            j=testd_parms(row,2);
        
            if std_pars(i,j) ~=0
                go_calculation=go_calculation+1;
            end
        end
 
        %go_calculation=2; % <--- For debug.
        if go_calculation == testd_rows
        
            for row=1:testd_rows
                i=testd_parms(row,1);
                j=testd_parms(row,2);
                table(i,5*j-4)=std_pars(i,j);
                disp(sprintf('%s = params(%d,%d) standard value = %f ',char(p_names(i,j)),i,j,std_pars(i,j) ) );
            end
 
            pass_exp=0;
            test_exp=max_power;
            fail_exp=max_power+1;
        end
        
        %Increment value to find maximum power of two that allows cycling
        last_failure=1;
        while (abs(pass_exp-fail_exp) > precision)
            
            disp(sprintf('Testing increments of'));
        
            for row=1:testd_rows
                i=testd_parms(row,1);
                j=testd_parms(row,2);
                disp(sprintf('     %s=params(%d,%d). So far: Pass, fail, test: %0.1f %0.1f %0.1f',char(p_names(i,j)),i, j,pass_exp, fail_exp, test_exp));
            end
 
            for row=1:testd_rows
                i=testd_parms(row,1);
                j=testd_parms(row,2);
                params(i,j)=std_pars(i,j)*(2^test_exp);
                disp(sprintf('%0.2f X increase in %s  :',params(i,j)/std_pars(i,j),char(p_names(i,j)) ) );
            end
            
            cycle=cell03_run_plot(init);
                                
            if cycle==1
                pass_exp=test_exp;
            end
 
            if cycle~=1
                 fail_exp=test_exp;
                 last_failure=cycle;
            end
 
            test_exp=(pass_exp+fail_exp)/2;
                    
            if (cycle==1) & (pass_exp >= max_power )
                fail_exp=pass_exp;
            end
        end
        
        %Set up output table in fives; in cols 4,9,14... the highest
        %working value for the parameter found; in cols 5, 10, 15 the
        %last failure criterion
        for row=1:testd_rows
            i=testd_parms(row,1);
            j=testd_parms(row,2);
            table(i,5*j-1)=std_pars(i,j)*(2^pass_exp);
            table(i,5*j)=last_failure;
            table(i,5*j-2)=2^pass_exp;
        end
        
        params=std_pars;
 
%Reset parameters to standard values
params=std_pars;
[i,j]=size(table);
disp(sprintf('table(%d,%d)',i,j));
 
disp('Output table: each row contains: Parameter name, std value, greatest possible decrement, failure criterion for closest failing decrement (if any), greatest possible increment, failure criterion for closest failing increment (if any)');
for row=1:rows
    for col=1:cols
        if table(row,5*col-4)~=0
            disp(sprintf('Parameter %s: %f %f %d %f %d',char(p_names(row,col)), table(row,5*col - 4), table(row,5*col - 3), table(row,5*col - 2), table(row,5*col - 1), table(row,5*col) ) );  
        end
    end
end
